# Binding continuous response features of extended movements: Integration with discrete response but not stimulus features

**DOI:** 10.1007/s00426-026-02295-5

**Published:** 2026-04-22

**Authors:** Anna Foerster, Birte Moeller, Maria Nemeth, Moritz Schaaf, Christian Frings, Roland Pfister

**Affiliations:** 1 Department of Psychology, Trier University, Universitätsring 15, 54296 Trier, Germany; 2 Institute for Cognitive and Affective Neuroscience, Trier University, Universitätsring 15, 54296 Trier, Germany

## Abstract

Binding and retrieval have been portrayed as key mechanisms in human action control. This theoretical framework posits that features of perception and action (e.g., object color, effector, etc.) are bound to each other. Afterwards, re-encountering one feature can retrieve bound features, thereby facilitating action repetition. Previous research focused on bindings with discrete response features (e.g., responding with a left or right button). This focus neglects the rich information required to control body movements. Here, we investigated continuous response features of extended movements related to both action goals and the implementation of these goals. Participants swiped their fingers on a tablet toward a small, briefly presented target circle that was accompanied by a sound. We analyzed response errors and similarity between successive response positions, depending on the sequence of (1) swiped hands, (2) irrelevant sounds, and (3) target positions. Sound repetitions did not bias responses toward preceding target or response positions whereas hand repetitions biased responding toward both positions, especially when target positions repeated. On the one hand, stimulus-response binding and retrieval seem to be remarkably focused on discrete response features, thus challenging the role of this mechanism in controlling overt body movements. On the other hand, fine-grained features of a response can be bound and retrieved from other response features, providing short-cuts to both the agents’ previous action goals and the implemented deviation from these goals.

Public significance statement

Humans do not explicitly think about every action. Instead, they automatically connect what they perceive with how they act, a process that facilitates repetition of actions. Yet, which aspects of perception and action can become connected remains a topic of debate. Our study shows that these connections may extend beyond categorical action features (e.g., pressing a certain button on a phone) to metric action features (e.g., touching a specific position on a screen).

**Supplementary Information:**

The online version contains supplementary material available at 10.1007/s00426-026-02295-5.

## Introduction

Short-cuts to responses are ubiquitous in action control. Different features of a (planned) action event are bound to each other (e.g., Frings et al., 2024; Frings et al., 2020; Henson et al., 2014; Hommel, 2004). That is, connections are formed that include characteristics of attended objects (e.g., color, sound, or shape) and responses (e.g., pressing or releasing a key with a finger or a foot). If agents find themselves in a similar situation later, the cognitive system retrieves bound features automatically. As demonstrated in numerous studies that targeted discrete response features, the activation of a response through retrieval facilitates its performance but hampers performance of other responses (e.g., Dutzi & Hommel, 2009; Hommel, 1998; Frings et al., 2007; Moeller et al., 2016; Stoet & Hommel, 1999; for a review, see, e.g., Frings et al., 2020).

**Fig. 1:**
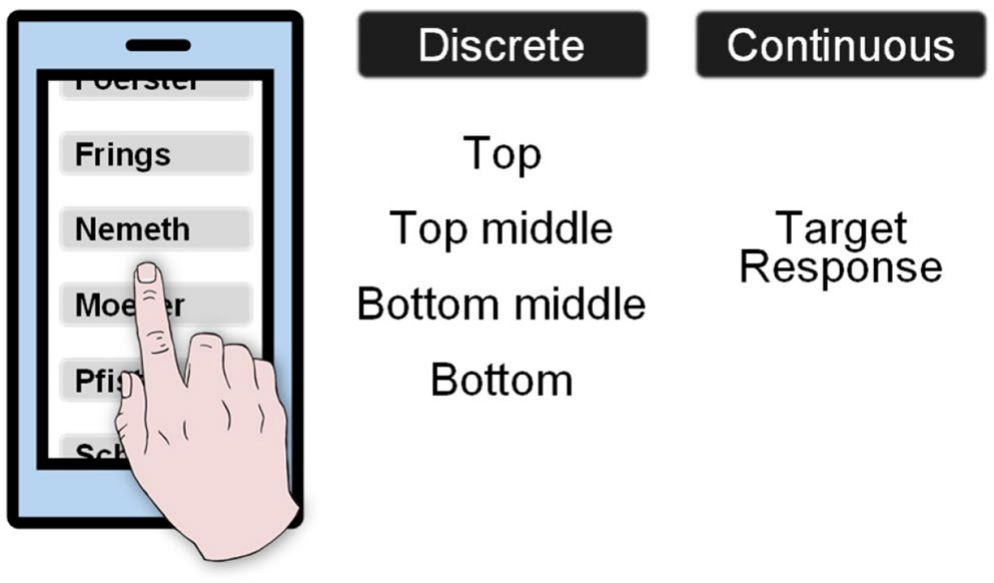
Discrete and continuous response features. Someone wants to call Maria Nemeth on their phone. However, they accidentally tap just below the designated button. Discrete features, distinguishing between categorical positions (i.e., top, top middle, etc.), would not capture the response adequately. Instead, continuous features relating to the goal (i.e., the target position) and the implemented deviation from this goal (i.e., the response position), describe the response more precisely. The central research questions of this experimental series were whether continuous response features are subject to binding and retrieval, and if so, whether the involved features relate to the goal, to the implemented deviation from this goal, or both.

Such discrete features specify the response in meaningful categories, for example which of six adjacent buttons should be pressed (see Fig. 1), and can be included in action plans (e.g., Stoet & Hommel, 1999; see also Rosenbaum, 1985). However, for the execution of a body movement, discrete response features do not suffice; rather, motor control also requires processing of continuous features that specify these movements (e.g., at which position the finger should press down and where it actually does). The current experimental series investigates whether continuous response features that are related to both action goals and the implementation of these goals are an integral part of binding and retrieval processes. The involvement of continuous response features would suggest that binding is a facet of precise action control.

Bindings can already be (partially) established before the involved responses are executed. For example, binding of action plans can be demonstrated by splitting up two tasks A and B into an ABBA sequence (e.g., Mocke et al., 2022; Stoet & Hommel, 1999; Wiediger & Fournier, 2008). In these experiments, participants planned a response for task A, then planned and executed task B, and only afterwards executed the pre-planned response for task A. If task B shared some but not all response features with task A, performance in task B was typically decreased, compared to when no features overlapped. These so-called partial overlap costs between task B and the (only planned) task A point to binding of planned response features. In other words, action plans are realized as bindings between response features, not only via the sum of the pre-activations of these features (Stoet & Hommel, 1999). Such feature integration therefore specifies the appropriate action amongst a plethora of possible but inappropriate actions in a situation.

Most studies investigated bindings for discrete response features for planned and executed responses. There is scarce evidence for binding and retrieval for continuous response execution features though. In multiple studies, researchers assessed response durations (i.e., the time interval between pressing and releasing a key) as a continuous feature of response execution (Pfister et al., 2023). More precisely, they computed the absolute difference between the response durations of successive responses in trial n-1 and trial n. Smaller differences therefore indicate more similarity in the execution of the two responses. Absolute response duration differences were sometimes smaller (i.e., similarity was sometimes larger) when stimuli repeated rather than changed (Bogon et al., 2023; Pfister et al., 2022; Varga et al., 2022, 2024; but see Foerster et al., 2022, 2023). This effect is in line with retrieval of the response duration from trial n-1 upon stimulus repetition in trial n. However, this modulation only emerged if the bound discrete stimulus feature was relevant because it indicated the required response. It did not emerge with irrelevant stimuli that were not assigned to any response. Further, the modulation was larger if response duration was relevant in the task, that is, if short and long keypresses were specifically instructed. The weak evidence for binding and retrieval of continuous response features in these studies may partly be due to the relatively confined response durations for these ballistic keypress responses.

A few studies suggest that continuous, spatial response features of more extended movements influence following movements. For example, reaching movements toward visible targets were biased toward recent target positions (Verstynen & Sabes, 2011; see also Marinovic et al., 2017), speaking for persistent activation of continuous actions. Another study suggested that task-relevant stimulus features bind and retrieve spatial features of movement trajectories (Moher & Song, 2013). In this study, participants had to reach towards a uniquely colored target and ignore two distracting targets which had the other possible color. Critically, the curvature of the movement trajectory depended on whether colors of the target and distractors repeated or swapped.

In the current study, participants swiped toward the position of rapidly vanishing targets, allowing us to assess binding and retrieval of continuous response features in such extended movements. We examined here more thoroughly whether continuous response features that are highly relevant for precise motor control are subject to binding and retrieval. That is, we disentangled for the first time continuous target positions (i.e., features of the response goal) from response positions (i.e., features of the implemented response) and scrutinized whether they are bound to discrete stimulus and response features.

**Fig. 2:**
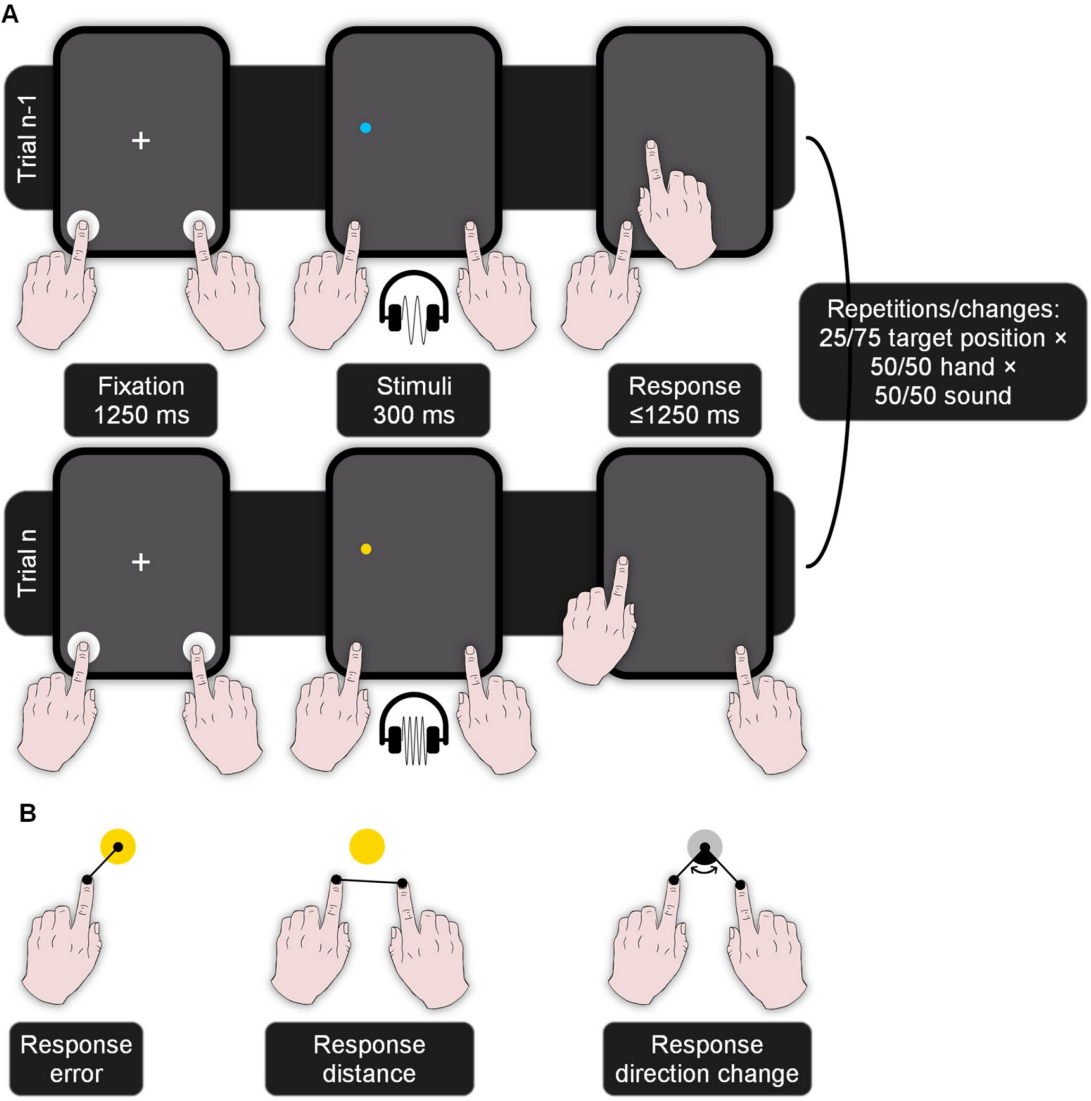
Sequential trial structure in Experiment 1 and dependent variables. **(A)** Participants had to touch two starting areas with their two index fingers during the presentation of the fixation cross. After 1250 ms, a target circle appeared for 300 ms, accompanied by one of two irrelevant sounds. The color of the target indicated with which hand participants had to swipe (here: blue = right, yellow = left). After the target onset, they had 1250 ms to swipe as closely to the position of the (already vanished) target as possible and lift their finger. In successive trials (trial n-1 and n), the target position repeated in 25% of the trials, the hand repeated in 50% of the trials, and the sound repeated in 50% of the trials. **(B)** The main dependent variables were the response error (the distance between the target and the response in trial n), the distance between the response in trial n and the response in trial n-1, and the response direction change between the response in trial n and the response in trial n-1. Binding and retrieval of sounds and target positions predict smaller response errors for sound repetitions than changes when the target position repeats. Binding and retrieval of sounds and executed responses predict more similar successive responses, that is, smaller distances and direction changes for sound repetitions than changes when the target position repeats.

Participants indicated the position of a small target circle on a tablet screen by swiping from a starting area to the assumed target position (see Fig. 2A). The target only flashed for a short time to exclude explicit knowledge about whether the target was hit in each trial. To provoke graded errors, the target appeared briefly, and the response window was short. Movements were therefore ballistic and probably automatic as soon as participants familiarized themselves with the task. In addition to the target position, we manipulated stimulus and response features. The color of the target indicated whether to use the index finger of the left or right hand for the swiping movement in Experiment 1 and 2.^1^ While the target color was thus task-relevant, it was not contingent on the target position. Further, each target was accompanied by a sound, which was irrelevant since its identity was chosen randomly and was not contingent on target color or position.

On the one hand, continuous aspects of the response goal (i.e., the target position) could be bound. Such bindings would entail that repetitions of the other features in successive trials retrieve the previous target position. For target position repetitions, a bias toward the former target position through retrieval could decrease the *response error*, that is, the distance between the target position and the response position (see Fig. 2B).

On the other hand, continuous aspects of the implemented response (i.e., the response position) could be bound. Such bindings would entail that repetitions of the other features retrieve the previous response position. A bias toward the former response position through retrieval could reduce the *response distance* between the current response position (trial n) and the previous response position (trial n-1; see Fig. 2B). It could further reduce the *response direction change*, that is, the (unsigned) angle between the current and the previous error direction (see Fig. 2B), at least in trials where the target position also repeats.

Binding and retrieval might also influence the initiation time, that is, the time between target onset and exiting the starting area, and the movement time, that is, the time between exiting the starting area and ending the movement (Wiediger & Fournier, 2008). Specifically, retrieval of continuous aspects of the response upon sound or hand repetitions could facilitate response initiation and/or execution and therefore speed up performance. While these measures can indicate binding and retrieval, they are not specific to the nature of the continuous response features involved, that is, whether they relate to the response goal or its implementation. Therefore, these temporal variables were considered secondary in all experiments and are reported in the *Supplementary Material*. Table 1 provides an overview of the five dependent variables and the features they capture.

**Table 1:** Dependent variables and the investigated features

Dependent variable	Investigated continuous features
Response error	Response goal (i.e., the target position)
Response distance	Implemented response (i.e., the response position)
Response direction change	Implemented response (i.e., the response position)
Initiation time	Response goal and/or implemented response
Movement time	Response goal and/or implemented response

## Experiment 1

### Introduction

In Experiment 1, we assessed binding of both the target and response position to an irrelevant, discrete stimulus feature accompanying the presentation of the target (one of two sounds). As these sounds were irrelevant for conducting the task and not contingent on task-relevant features, bindings including these features are likely implicit and can be studied independently from established task rules. We specifically focused on sequences with target position repetitions because these sequences are diagnostic about whether features of the response goal or the implemented response are retrieved.

On the one hand, if the sound retrieves the previous target position (i.e., the response goal), this should manifest in smaller response errors for sound repetitions than changes, whereas it should not affect response distance and direction change. On the other hand, if the sound retrieves the previous response position (i.e., the implemented response), this should manifest in smaller response distances and direction changes for sound repetitions than changes, whereas it should not affect response errors. Finally, if both the previous target and the response position are bound to and retrieved by the sound, the described effects should emerge in all three main measures. We further assessed the effector-specificity of these binding and retrieval effects via the comparison of sequences where the same or a different hand had to be used (Moeller et al., 2015; for evidence for abstract trajectory representations, see Wong et al., 2019).

As secondary analyses, initiation and movement time were investigated, although these measures cannot be unequivocally mapped to different response features. Further, for all five dependent measures, we explored whether binding and retrieval effects are modulated by the size of the response error during binding (i.e., in the previous trial n-1). For brevity, we report these secondary analyses in the *Supplementary Material*.

### Method

#### Participants

For categorical, erroneous responses, previous binding and retrieval effects between irrelevant stimulus features and goal-related response features were *d*_*z*_ = 0.40 in response times (Foerster et al., 2021). At α = 5%, a sample of 51 participants has a power of 1 – β ≥ 80% to detect this effect size in a two-tailed test (computed with the *power.t.test* function in *R* version 4.3.1; R Core Team, 2023). To allow for counterbalancing, we preregistered to collect 52 analyzable datasets.

We preregistered to invite pilot samples of eight participants each, respectively. Our goal was to exclude a maximum of 25% of participants from such a pilot sample (see *Data treatment*) and to adapt the study design (e.g., response deadlines or the length of the experiment) and invite a new pilot sample if we did not meet this goal. In the first pilot version of this experiment, exclusions were higher than 25% (three out of nine participants excluded; the experimenters invited nine instead of eight participants) so that we adapted the study design (see *Procedure).* The second pilot version of the experiment did not exceed the exclusion threshold, and therefore, the data collection continued according to the power analysis above.

Four participants had to be excluded and replaced based on our preregistered criteria (see *Data treatment*). The final sample consisted of 52 participants with a mean age of 23.2 years (*SD* = 3.0 years). Thirty-seven of these participants identified as female, 14 as male, and one as non-binary. Three reported themselves to be left-handed, 49 to be right-handed, and nobody to be ambidextrous.

#### Apparatus and stimuli

The experiment was conducted on tablets (iPad Pro, 6th generation) in portrait mode. The application had a resolution of 1366 × 1024 pixels and was upscaled to fill the whole 12.9-inch screen. Participants wore headphones.

Participants placed their left and right hands on two starting areas in the bottom left and bottom right corners of the screen (see Fig. 2A). The starting areas were 100 pixels in diameter and their centers were 100 pixels away from the screen borders. Target circles were 20 pixels in diameter and appeared in a position that was (a) at least 100 pixels away from the left and right screen borders, (b) at least 150 pixels away from the top screen border, and (c) at least 300 pixels away from the bottom screen border. The target was always colored in either blue or yellow, and this color indicated with which hand participants had to swipe. The assignment of colors to hands was counterbalanced across participants. The irrelevant sound (400 vs. 800 Hz) was uncorrelated with both the position and the color of the target.

#### Procedure

Participants were instructed to try to hit the position of the target as quickly and accurately as possible. In the background of the instruction text, participants saw the starting areas and a target. They learned that the starting areas would be in the same position throughout the entire experiment. The instructions specified that the target would appear in a random position, that its color would indicate which hand to use, and that one of two irrelevant sounds would play simultaneously. Participants should not allow themselves to be distracted by hearing these sounds. Next, they received a detailed description of the procedure of a trial: First, they had to touch the starting areas with both index fingers. As soon as a target appeared, they had to move their left (e.g., yellow target) or right hand (e.g., blue target) as fast and accurately as possible to the position of the target. To indicate the final position, they had to lift their finger (e, g., Wirth et al., 2020). Finally, they had to return to the starting area as fast as possible. Participants were free to ask questions about the task.

The experiment had twelve blocks. The first block was considered practice and had 33 trials. The remaining eleven blocks had 81 experimental trials each. In the first trial of each block, the position and color of the target as well as the sound were chosen randomly. In the remaining trials of the block, the target position was random in 75% of the trials (target position change). In the other 25% of the trials, the target position was the same as in the preceding trial (target position repetition). The target color, which indicated the required hand, repeated in 50% of the trials and changed in the other 50% of the trials. Similarly, the irrelevant sound pitch repeated in 50% of the trials and changed in the other 50% of the trials. Therefore, there were eight possible combinations of target position sequence, hand sequence, and sound sequence. The four sequence combinations with target position repetitions occurred five times in each experimental block (and two times in the practice block). The four sequence combinations with target position changes occurred fifteen times in each experimental block (and six times in the practice block).

A trial began with the presentation of a white fixation cross for 1250 ms (see Fig. 2A). The colored target then appeared for 300 ms and a sound played for the same duration, after which the screen went blank. Participants had to touch both starting areas during fixation. After target onset, they had to swipe to the target and lift their finger to indicate the target position. This response had to be finished within 1250 ms after target onset. A trial was successful if participants touched both starting areas during fixation, swiped with the correct hand, and lifted the finger outside its starting area within the response deadline, while the finger of the other hand stayed on its starting area.

If participants erred, they received an error message for 2000 ms in red font. There were different types of errors: (1) participants began a touch outside of a starting area (German message: “Start nicht getroffen!”, translation: “Start not hit!”), (2) they did not touch both starting areas before target onset (German message: “Schneller zurück zum Start!”, translation: “Faster back to the start!”), (3) they swiped out of a starting area before target onset (German message: “Zu früh gestartet!”, translation: “Started too early!”), (4) they lifted their finger from the screen before target onset (German message: “Zu früh beendet!”, translation: “Ended too early!”), (5) they swiped out of the wrong starting area after target onset (German message: “Falscher Finger!”, translation: “Wrong finger!”), (6) they swiped the second finger out of its starting area or lifted it from the screen (German message: “Behalte den anderen Finger auf der Startfläche!”, translation: “Keep the other finger on the starting area!”), (7) they lifted their finger from the screen after target onset within a starting area (German message: “Bitte Finger zum Zielkreis bewegen!”, translation: “Please move finger toward target circle!”), (8) they did not lift their finger from the screen within the response deadline after leaving a starting area (German message: “Beende deine Bewegung zügiger!”, translation: “Finish your movement faster!”).

Participants did not receive immediate feedback about their response error (i.e., the deviation of their response position from the target position). However, after each block, participants were informed about the percentage of successful movements with the correct hand and the mean response error. Participants were again encouraged to hit the target as fast and precisely as possible. They continued with the next block after a self-paced break.

### Results

#### Software

We processed and analyzed the data in *R* 4.4.1 (R Core Team, 2023) with the *R* packages *data.table* version 1.15.4 (Barret et al., 2024), *ez* version 4.4-0 (Lawrence, 2016), *mousetRajectory* version 0.2.1 (Pfister et al., 2024), *schoRsch* version 1.10 (Pfister & Janczyk, 2016), *tidyverse* version 2.0.0 (Wickham et al., 2019), and *viridis* version 0.6.5 (Garnier et al., 2024). These packages were version-controlled via the *R* package *groundhog* version 3.2.0 (Simonsohn & Gruson, 2024), set to the newest versions available on July 1st, 2024. We calculated the outlier measure with code that we translated from MATLAB (Jones, 2019).

#### Data treatment

We excluded the practice block and the first trial of each experimental block. Errors were defined as trials in which participants did not touch both starting areas during fixation, began touching the screen outside both starting areas, lifted their finger from the screen in the starting area, swiped out of a starting area before the onset of the target, swiped the wrong hand out of its starting area, or did not lift their finger from the screen within the response deadline. We excluded and replaced three participants who committed more than 40% errors. For the remaining participants, we excluded trials with errors (on average: 13.50%) and trials after errors (12.70%).

For the remaining correct trials, we identified trials with low precision, defined as trials with response errors greater than 200 pixels. We excluded and replaced one participant who had more than 40% trials with low precision. For the remaining participants, we excluded trials with low precision (1.82%). We selected only trials with the same target position as the preceding trial (25.00%).

Finally, we identified and excluded outlier trials (4.97%), defined as trials where the median distance of the initiation time or of the movement time from all other data points was greater than 3 Sn (computed separately for each participant and design cell; Rousseeuw & Croux, 1993). All participants delivered at least 10 observations in each design cell after these exclusions and were included in the analyses.

We report the effect size partial eta-squared (η_p_^2^) for analyses of variance (ANOVAs), which are interpreted as small starting from η_p_^2^ = 0.01, as medium starting from η_p_^2^ = 0.06, and as large starting from η_p_^2^ = 0.14 (Cohen, 1988).

#### Main analyses

We analyzed our three main dependent variables, that is, response error, response distance, and response direction change in 2 × 2 ANOVAs with the within-subjects factors hand sequence (repetition vs. change) × sound sequence (repetition vs. change). Significant two-way interactions were followed by two-tailed, paired-samples *t*-tests which compared sound repetitions versus sound changes separately for hand repetitions and changes.^2^

**Fig. 3:**
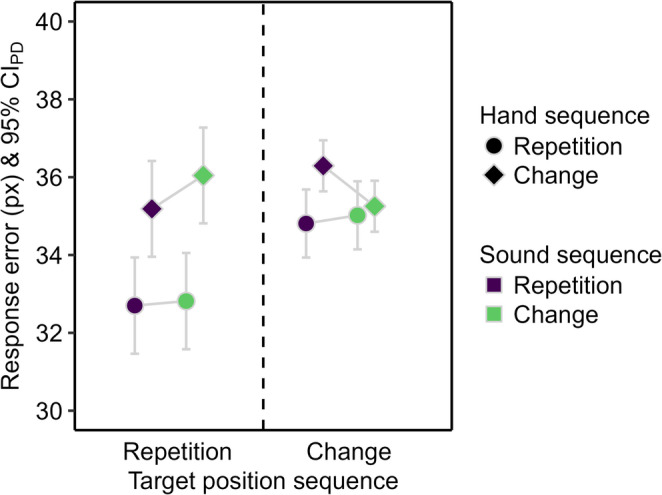
Descriptive statistics for the response error in Experiment 1. Mean response error in pixels (px). Means are depicted as a function of sound repetition (purple) and change (green), hand repetition (circle) and change (diamond), and target position repetition (left) and change (right). The error bars are 95% confidence intervals of paired differences (CI_PD_), visualizing two-tailed *t*-tests between sound sequences.

The response error was lower for hand repetitions than for hand changes (see Fig. 3), *F*(1, 51) = 18.26, *p* < .001, η_p_^2^ = 0.26. The main effect of sound sequence and the two-way interaction were not significant, *F* ≤ 1.52, *p* ≥ .223, η_p_^2^ ≤ 0.03.

**Fig. 4:**
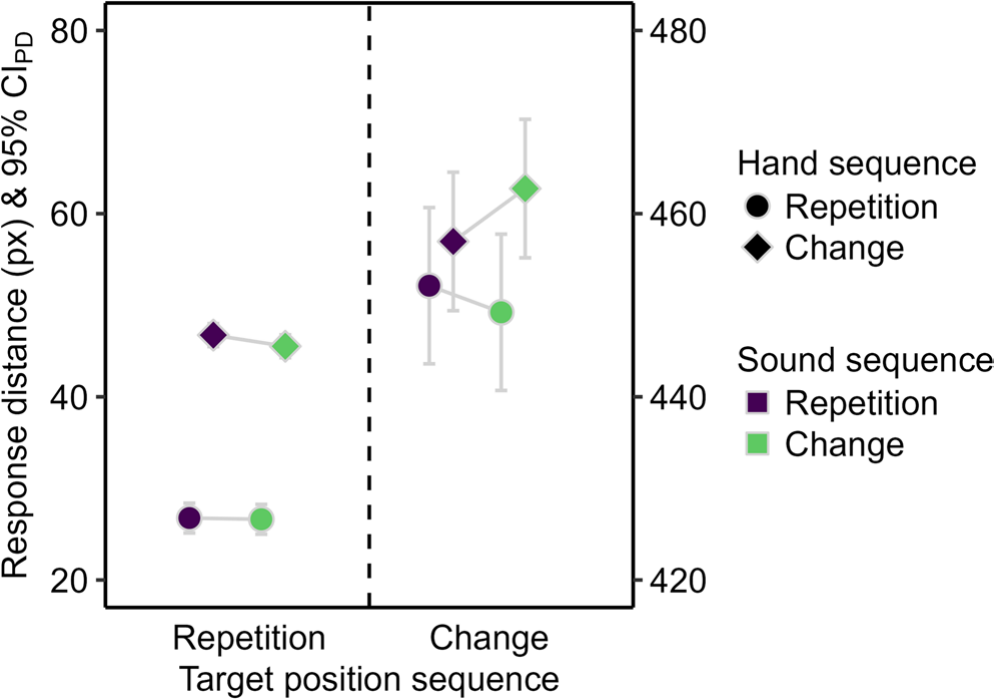
Descriptive statistics for the response distance in Experiment 1. Mean response distance in pixels (px). Means are depicted as a function of sound repetition (purple) and change (green), hand repetition (circle) and change (diamond), and target position repetition (left) and change (right). The error bars are 95% confidence intervals of paired differences (CI_PD_), visualizing two-tailed *t*-tests between sound sequences. Changing the target position inevitably resulted in longer response distances than repeating the target position. Therefore, target position sequences are mapped to separate y-axes to facilitate the comparison of sound and hand sequence effects.

The response distance was shorter for hand repetitions compared to hand changes (see Fig. 4), *F*(1, 51) = 44.74, *p* < .001, η_p_^2^ = 0.47. The main effect of sound sequence and the two-way interaction were not significant, *F* ≤ 1.77, *p* ≥ .189, η_p_^2^ ≤ 0.03.

**Fig. 5:**
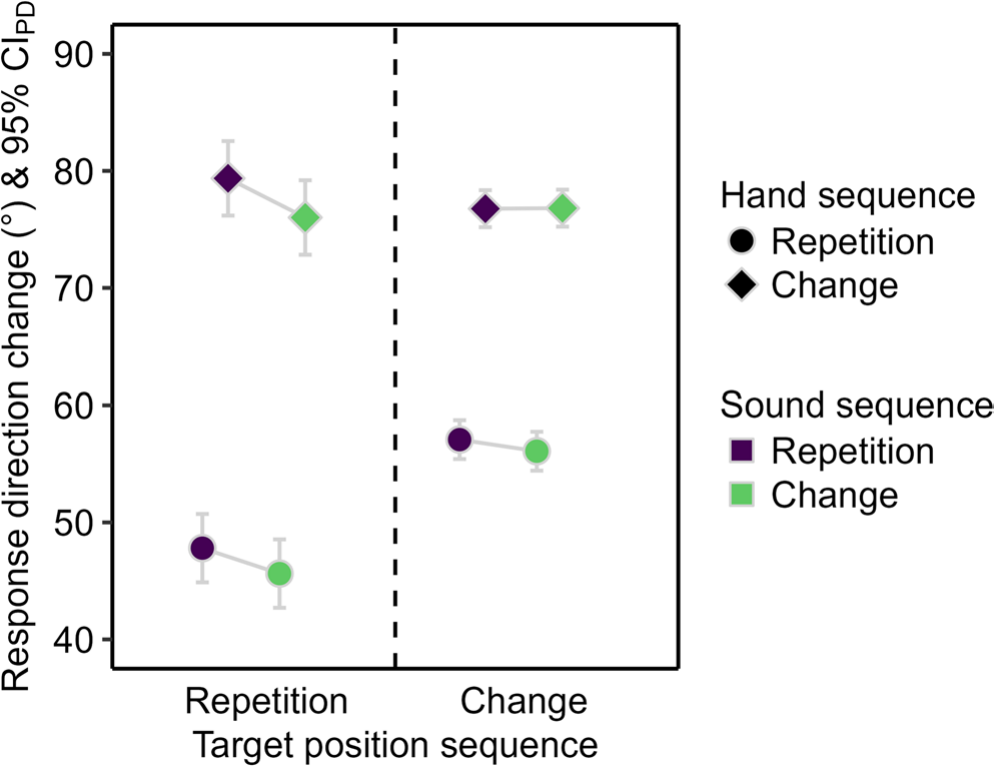
Descriptive statistics for the response direction change in Experiment 1. Mean (absolute) response direction change in degrees (°). Means are depicted as a function of sound repetition (purple) and change (green), hand repetition (circle) and change (diamond), and target position repetition (left) and change (right). The error bars are 95% confidence intervals of paired differences (CI_PD_), visualizing two-tailed *t*-tests between sound sequences.

The response direction change was smaller for hand repetitions than changes (see Fig. 5), *F*(1, 51) = 158.38, *p* < .001, η_p_^2^ = 0.76, and for sound changes than repetitions, *F*(1, 51) = 7.51, *p* = .008, η_p_^2^ = 0.13. The interaction of the two factors was not significant, *F* < 1.

### Discussion

In Experiment 1, participants had to swipe to a briefly presented target with their left or right hand. The color of the target indicated which hand to use, and one of two irrelevant sounds accompanied the target presentation. We hypothesized that the identity of the irrelevant sound stimulus could be bound to and then retrieve continuous response features.

However, our main analyses did not support binding and retrieval between irrelevant, discrete stimulus features and continuous response features: When the target position repeated, sound repetitions did not reduce the response error, response distance, or response direction change relative to sound changes. In other words, when the target position repeated, sound repetitions biased responses toward neither the preceding target position nor the preceding response position. Rather, for direction changes, we observed the opposite-than-predicted data pattern, as direction changes were higher for sound repetitions than for sound changes. Further, these null and reversed effects for the irrelevant sound sequence were modulated by neither hand sequence nor the size of the response error during binding (i.e., the size of the response error in trial n-1).

In stark contrast, the secondary analyses of the temporal variables, especially in the initiation time, point to binding and retrieval that involves irrelevant, discrete stimulus features (see *Supplementary Material*). However, as there was no evidence for retrieval of the response position and only weak evidence for retrieval of the target position in the appropriate spatial measures, it seems unlikely that retrieval of these continuous response features is responsible for the sequential effects observed in the initiation time. Rather, it seems like the irrelevant sound entered a binding with the required hand, as we observed strong partial repetition costs in initiation times, indicative of a slowdown of responding whenever one feature changed.

## Exploratory re-analyses of Experiment 1

### Introduction

Our initial focus for Experiment 1 was only on sequences with target position repetitions because we had clear predictions for sound repetition benefits to emerge. Nonetheless, we planned separate, secondary analyses for target position changes. Instead of assessing target position changes separately from target position repetitions though, we decided to conduct an analysis of the impact of target position, sound, and hand sequence.

This approach allows for an assessment of the processes that might underlie the observed influences of hand sequence. One explanation is that hand repetitions trigger retrieval of previously bound continuous response features, reducing the response error, distance and direction change compared to hand changes. The other explanation is that practice is at work when using the same hand successively instead of changing it. Practice could manifest in two ways. First, the translation of a target position into an appropriate movement might be easier, reducing the response error. Second, movements might be more similar, reducing response distance and direction change without an actual specification of erroneous aspects of the movement. Crucially, binding and practice can be disentangled by comparing the hand sequence effect between target position repetitions and changes. Practice would predict a similar effect of hand sequence after both target position repetitions and changes, while binding and retrieval of specific response features (related to the goal and its implementation) would predict a stronger effect of hand sequence for target repetitions than changes. The full predictions for this design can be found in the *Introduction* of Experiment 2, for which the following analysis was preregistered.

### Results

We identified and excluded outliers as described above for these additional trials with target position changes (exclusion of 5.28% of the trials) and analyzed all five dependent variables in 2 × 2 × 2 ANOVAs with the within-subjects factors target position sequence (repetition vs. change) × hand sequence (repetition vs. change) × sound sequence (repetition vs. change). In case of significant three-way interactions, we conducted a 2 × 2 ANOVA only for target position changes (for results on target position repetitions, see *Main analyses* in *Experiment 1*). Significant two-way interactions were followed by separate two-tailed, paired-samples *t*-tests. For brevity, we report the results on the initiation and movement time in the *Supplementary Material*.

The response error was smaller for target position repetitions than changes (see Fig. 3), *F*(1, 51) = 15.28, *p* < .001, η_p_^2^ = 0.23, and for hand repetitions than changes, *F*(1, 51) = 17.27, *p* < .001, η_p_^2^ = 0.25. The main effect of sound sequence was not significant, *F* < 1. The two-way interaction between target position and hand sequence was significant, *F*(1, 51) = 9.64, *p* = .003, η_p_^2^ = 0.16, because the reduction of the response error for hand repetitions compared to changes was larger for target position repetitions, *t*(51) = 4.27, *p* < .001, *d*_*z*_ = 0.59, than changes, *t*(51) = 2.14, *p* = .037, *d*_*z*_ = 0.30. The two-way interaction between target position and sound sequence was also significant, *F*(1, 51) = 4.36, *p* = .042, η_p_^2^ = 0.08, because sound repetitions descriptively reduced the response error compared to sound changes when the target position repeated, *t*(51) = 1.23, *p* = .223, *d*_*z*_ = 0.17, but descriptively increased the response error when the target position changed, *t*(51) = -1.57, *p* = .122, *d*_*z*_ = -0.22. The remaining two-way interaction between hand and sound sequence, *F* < 1, and the three-way interaction, *F*(1, 51) = 2.98, *p* = .090, η_p_^2^ = 0.06, were not significant.

The response distance was shorter for target position repetitions than changes (see Fig. 4), *F*(1, 51) = 34524.37, *p* < .001, η_p_^2^ > 0.99, and for hand repetitions than changes, *F*(1, 51) = 40.63, *p* < .001, η_p_^2^ = 0.44. The main effect of sound sequence was not significant, *F* < 1. The two-way interaction between target position and hand sequence was significant, *F*(1, 51) = 9.17, *p* = .004, η_p_^2^ = 0.15, because the reduction of the response distance for hand repetitions compared to changes was larger for target position repetitions, *t*(51) = 6.69, *p* < .001, *d*_*z*_ = 0.93, than changes, *t*(51) = 3.38, *p* = .001, *d*_*z*_ = 0.47. The remaining two-way interactions, *F* ≤ 2.18, *p* ≥ .146, η_p_^2^ ≤ 0.04, and the three-way interaction were not significant, *F*(1, 51) = 3.32, *p* = .074, η_p_^2^ = 0.06.

The response direction change was smaller for target position repetitions than changes (see Fig. 5), *F*(1, 51) = 35.23, *p* < .001, η_p_^2^ = 0.41, for hand repetitions than changes, *F*(1, 51) = 129.82, *p* < .001, η_p_^2^ = 0.72, and for sound changes than repetitions, *F*(1, 51) = 8.17, *p* = .006, η_p_^2^ = 0.14. The interaction of target position and hand sequence was significant, *F*(1, 51) = 88.79, *p* < .001, η_p_^2^ = 0.64, because the reduction of the response direction change for hand repetitions compared to changes was larger for target position repetitions, *t*(51) = 12.58, *p* < .001, *d*_*z*_ = 1.75, than changes, *t*(51) = 9.34, *p* < .001, *d*_*z*_ = 1.29. The remaining two-way interactions, *F* ≤ 3.95, *p* ≥ .052, η_p_^2^ ≤ 0.07, and the three-way interaction were not significant, *F* < 1.

### Discussion

The exploratory comparisons between target position repetitions and changes hinted at binding and retrieval between irrelevant, discrete stimulus features and continuous response features of the goal. The target position and sound sequence interacted in the response error, reflecting a descriptive sound repetition benefit for target position repetitions but descriptive costs for changes. This pattern can be taken as tentative evidence for retrieval of the preceding target position through sound repetitions, decreasing the response error for target position repetitions but increasing it for target position changes. However, since this analysis was post-hoc and only not highly significant (*p* = .042), we consider the results of Experiment 2 before drawing any conclusions.

Both the main effects of hand sequence in the main analyses as well as the interaction of target position and hand sequence in the exploratory analyses are in line with the notion of binding and retrieval between discrete response features and continuous response features: The response error, response distance, and direction change were smaller when the hand repeated rather than changed, and these reductions were consistently larger for target position repetitions than for target position changes. This differential reduction cannot easily be explained by the notion that repeatedly using the same hand results in generally smaller errors (independent of the target position) and increases the similarity of the two successive movements (without actual specificity in the response position). Thus, it seems like hand repetitions retrieve previously bound target positions (reduced response error) and response positions (reduced response distance and direction change) specifically and bias performance toward these positions.

In sum, the results of Experiment 1 do not allow us to draw strong conclusions about the existence of binding and retrieval of continuous response features. For one, the analyses yielding positive evidence were exploratory. Second, Experiment 1 confounded target color sequences with hand sequences. As such, a viable alternative interpretation for the observed binding and retrieval between discrete response features and continuous response features is that this data pattern reflects binding and retrieval between task-relevant, discrete stimulus features (the target color) and continuous response features.

## Experiment 2

### Introduction

Experiment 2 served two purposes. First, it controlled for potential influences of target color sequences. To this end, we used two different pairs of colors to instruct the left or right hand in odd and even trials. With this manipulation, the color always changed between trials even when participants used the same hand. Second, we implemented the exploratory analyses of Experiment 1 as preregistered main analyses. To equalize the number of trials in each design cell of this analysis, the trial sequences now consisted of 50% target position repetitions and 50% changes.

With this additional design factor, the predictions are more complex than in Experiment 1: Binding between discrete stimulus features and continuous features of the response goal would entail that a sound repetition retrieves the preceding target position. As such, sound repetitions should reduce the response error compared to sound changes, although this benefit should only occur when the target position repeats, not when it changes. This binding should not affect the response distance and direction change. Binding between discrete stimulus features and implemented response features instead predicts that a sound repetition retrieves the preceding response position, irrespective of how close or far this response was from the target. As such, sound repetitions should reduce the response distance and direction change compared to response changes. This binding should not affect the response error. If both the target and the response position are bound to and retrieved from the sound, the described effects should emerge in all three measures. Sequential effects of sound sequence could be larger when the target position repeats than changes and when the hand repeats than changes.

Similar predictions apply for binding with discrete response features. Binding of features of the response goal would entail that a hand repetition retrieves the preceding target position. As such, the response error should be smaller for hand repetitions than changes whereas the response distance and direction change should not be affected. Binding of implemented response features predicts that a hand repetition retrieves the preceding response position. As such, the response distance and direction change should be smaller for hand repetitions than changes whereas response error should not be affected. If both binding processes are at work, we should find the described effects in all three measures. The interaction of hand and target position sequence is crucial for our interpretation of the results in terms of binding and retrieval. If the main effect of hand sequence would emerge independently of the target position sequence, the underlying processes could be related to practice. For one, movements toward targets might be more precise when using the same hand (independent of the preceding target position). Second, successive movements might be more similar when using the same than a different hand (without specifications of the final response position). However, a larger hand sequence effect for target position repetitions would strongly suggest that the actual target and response positions are bound to and retrieved from the hand.

For brevity, we again report the secondary analyses on initiation and movement time in the *Supplementary Material*.

### Method

#### Participants

In Experiment 1, we observed effects for significant interactions of target position sequence × hand sequence of *d*_*z*_ ≥ 0.42. Significant main effects of sound sequence or interactions with this factor showed smaller effect sizes with *d*_*z*_ ≥ 0.29. We collected 96 analyzable datasets to have a power of 80% (α = 5%) to detect the smaller effect size in a two-tailed test (computed with the *power.t.test* function in *R* version 4.3.1) and this sample size allows for counterbalancing. As in Experiment 1, we planned to do pilot studies with eight participants. As our first pilot study met our preregistered criteria, we filled up the sample according to the power analysis.

We conducted the experiment with 105 participants because we had to exclude the datasets from nine participants (see *Data treatment*). The 96 participants who entered our analysis had a mean age of 25.2 years (*SD* = 5.2 years). Seventy-five participants identified as female, 20 as male, and one as non-binary. Eight participants reported themselves to be left-handed, 86 to be right-handed, and two to be ambidextrous.

#### Apparatus, stimuli, and procedure

Apparatus, stimuli, and procedure of Experiment 2 were as in Experiment 1, except for the following changes:

First, we used four colors to indicate which hand to use (green, yellow, pink, and blue), which allowed us to disentangle color and hand sequence by presenting one color pair in odd trials and the other color pair in even trials. The assignment of the colors to the hands and to odd or even trials was fully counterbalanced across participants (4! = 24 combinations)^3^. Therefore, the target color always changed between trials, while the required hand could either repeat or change. In each trial, the current mapping of colors to hands was indicated by displaying the starting areas in the color corresponding to the associated hand.^4^

Second, the target position now changed in 50% of the trials and repeated in the other 50% of the trials: Again, in the first trial of each block, the position and the color of the target as well as the sound were chosen randomly (but the color was chosen from the two colors that were presented in odd trials). In the remaining trials of the block, all eight combinations of target position sequence, hand sequence, and sound sequence repeated ten times in experimental blocks (and four times in the practice block).

### Results

#### Data treatment

One participant aborted the experiment and was excluded and replaced.^5^ Our exclusion criteria were the same as for Experiment 1, but we did not exclude trials with target position changes^6^. We excluded the first block and the first trial of each following block. We excluded and replaced participants who committed more than 40% errors (seven participants). We excluded erroneous trials for the remaining participants (17.10%) and the trials immediately following these errors (16.20%). We excluded and replaced one participant who had more than 40% trials with low precision. For the remaining participants, we excluded trials with low precision (2.33%). We then excluded outlier trials (5.20%). All participants delivered at least 10 observations in each design cell after these exclusions and were included in the analyses.

#### Main analyses

The exploratory data analyses of Experiment 1 now served as our preregistered main analyses. Thus, we analyzed the response error, the response distance, and the response direction change in separate 2 × 2 × 2 ANOVAs with the within-subjects factors target position sequence (repetition vs. change) × hand sequence (repetition vs. change) × sound sequence (repetition vs. change). In case of significant three-way interactions, we conducted separate 2 × 2 ANOVAs for target position repetitions and changes. Significant two-way interactions were followed by two-tailed, paired-samples *t*-tests.

**Fig. 6:**
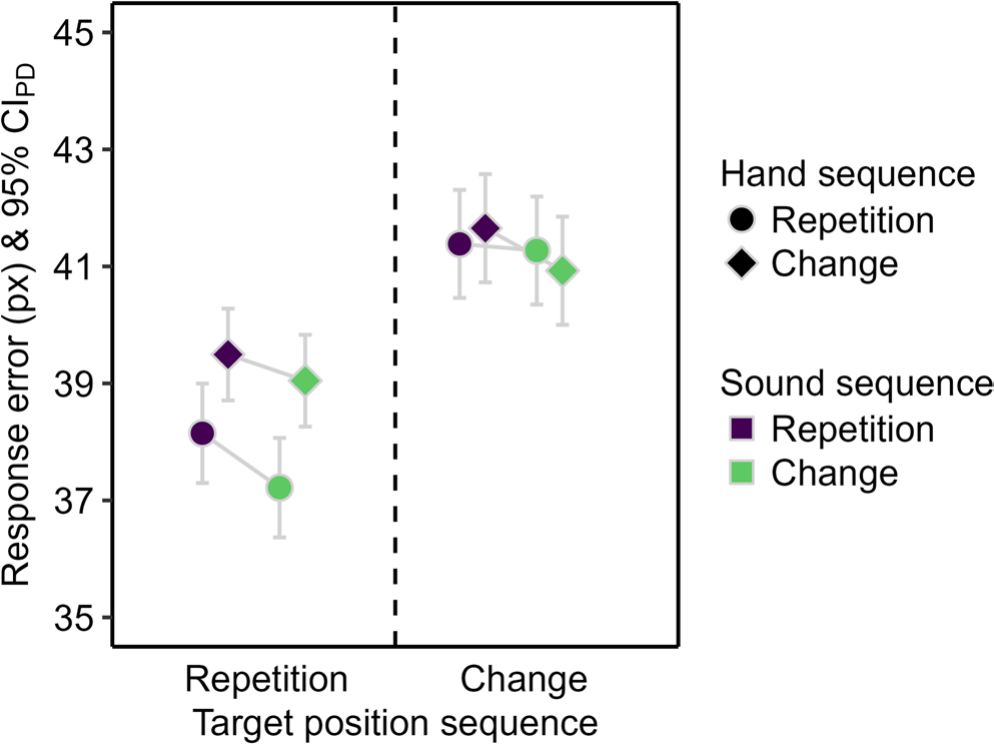
Descriptive statistics for the response error in Experiment 2. Mean response error in pixels (px). Means are depicted as a function of sound repetition (purple) and change (green), hand repetition (circle) and change (diamond), and target position repetition (left) and change (right). The error bars are 95% confidence intervals of paired differences (CIPD), visualizing two-tailed t-tests between sound sequences

The response error was smaller for target position repetitions than changes (see Fig. 6), *F*(1, 95) = 108.06, *p* < .001, η_p_^2^ = 0.53, and for sound changes than repetitions, *F*(1, 95) = 6.35, *p* = .013, η_p_^2^ = 0.06. The main effect of hand sequence was not significant, *F*(1, 95) = 3.78, *p* = .055, η_p_^2^ = 0.04. The two-way interaction between target position and hand sequence was significant, *F*(1, 95) = 17.55, *p* < .001, η_p_^2^ = 0.16, because hand repetitions reduced the response error relative to hand changes only for target position repetitions, *t*(95) = 3.18, *p* = .002, *d*_*z*_ = 0.32, not for target position changes, |*t*| < 1. The remaining two-way interactions were not significant, *F* < 1. The three-way interaction was also not significant, *F*(1, 95) = 1.84, *p* = .178, η_p_^2^ = 0.02.

**Fig. 7:**
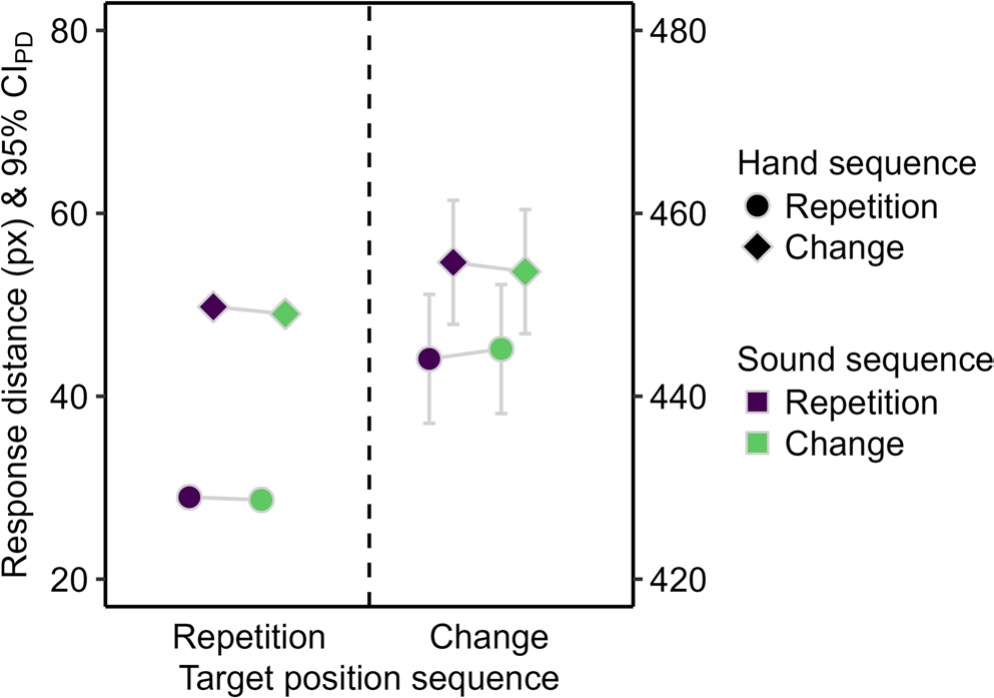
Descriptive statistics for the response distance in Experiment 2. Mean response distance in pixels (px). Means are depicted as a function of sound repetition (purple) and change (green), hand repetition (circle) and change (diamond), and target position repetition (left) and change (right). The error bars are 95% confidence intervals of paired differences (CIPD), visualizing two-tailed t-tests between sound sequences. Changing the target position inevitably resulted in longer response distances than repeating the target position. Therefore, target position sequences are mapped to separate y-axes to facilitate comparison of sound and hand sequence effects

The response distance was shorter for target position repetitions than changes (see Fig. 7), *F*(1, 95) = 35068.10, *p* < .001, η_p_^2^ > 0.99, and for hand repetitions than changes, *F*(1, 95) = 86.14, *p* < .001, η_p_^2^ = 0.48. The main effect of sound sequence was not significant, *F* < 1. The two-way interaction between target position and hand sequence was significant, *F*(1, 95) = 9.84, *p* = .002, η_p_^2^ = 0.09, because the reduction of the response distance for hand repetitions compared to changes was larger for target position repetitions, *t*(95) = 12.67, *p* < .001, *d*_*z*_ = 1.29, than changes, *t*(95) = 3.20, *p* = .002, *d*_*z*_ = 0.33. The remaining two-way interactions and the three-way interaction were not significant, *F* < 1.

**Fig. 8:**
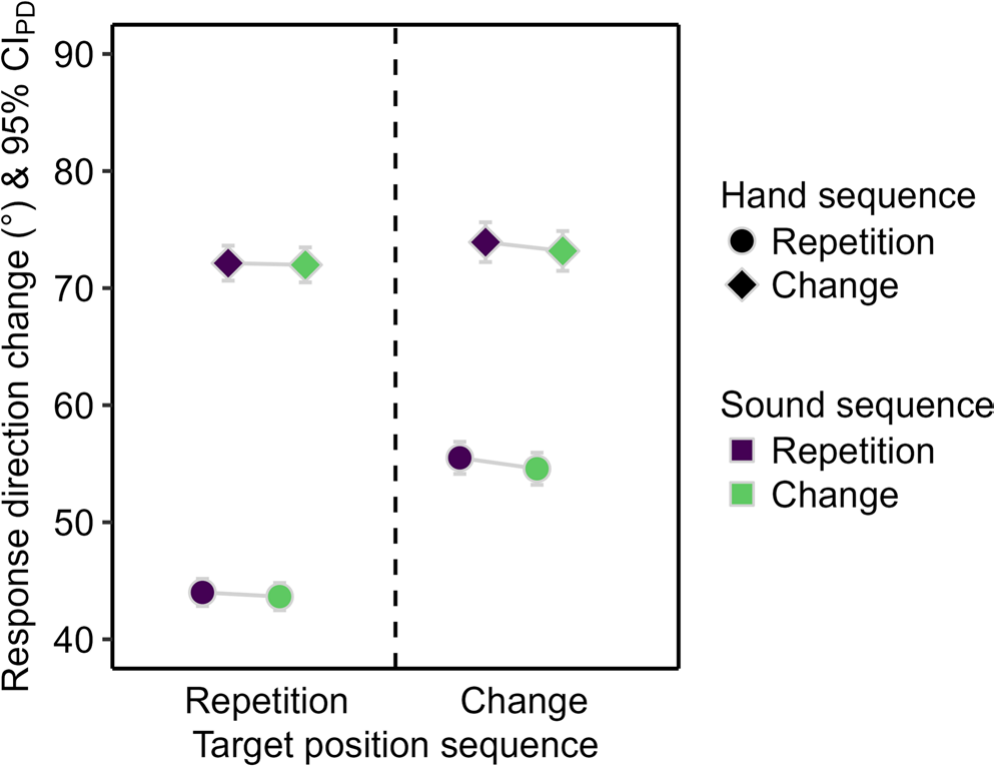
Descriptive statistics for the response direction change in Experiment 2. Mean (absolute) response direction change in degrees (°). Means are depicted as a function of sound repetition (purple) and change (green), hand repetition (circle) and change (diamond), and target position repetition (left) and change (right). The error bars are 95% confidence intervals of paired differences (CIPD), visualizing two-tailed t-tests between sound sequences

The response direction change was smaller for target position repetitions than changes (see Fig. 8), *F*(1, 95) = 160.47, *p* < .001, η_p_^2^ = 0.63, and for hand repetitions than changes, *F*(1, 95) = 318.98, *p* < .001, η_p_^2^ = 0.77. The main effect of sound sequence was not significant, *F*(1, 95) = 2.45, *p* = .121, η_p_^2^ = 0.03. The interaction of target position and hand sequence was significant, *F*(1, 95) = 155.58, *p* < .001, η_p_^2^ = 0.62, because the reduction of the response direction change for hand repetitions compared to changes was larger for target position repetitions, *t*(95) = 20.92, *p* < .001, *d*_*z*_ = 2.14, than changes, *t*(95) = 13.40, *p* < .001, *d*_*z*_ = 1.37. The remaining two-way interactions and the three-way interaction were not significant, *F* < 1.

### Discussion

Experiment 2 served to replicate the exploratory analyses of Experiment 1 and to rule out that the influences of hand repetitions stemmed from color repetitions.

Overall, the data of Experiment 2 do not support binding and retrieval between discrete stimulus features and continuous response features. Although the post-hoc analyses in Experiment 1 indicated an interaction of target position and sound sequence in response error, Experiment 2 did not replicate this critical pattern. Instead, Experiment 2 found sound repetition *costs* in the response error across target position sequences. If there was binding of the response goal to the sound, we would expect sound repetition *benefits* for target position repetitions.

The data of Experiment 2 provide clear evidence for binding and retrieval between discrete and continuous response features. In line with Experiment 1, the reduction of the response error, distance and direction change through hand repetitions was consistently larger for target position repetitions than changes. The response error was not affected by hand sequence when the target position changed. This result again supports the assumption that hand repetitions retrieve previously bound target and response positions.

The analysis of the temporal variables gives again some indication for binding and retrieval involving discrete features (see *Supplementary Material*). The results of the initiation time in both experiments suggest binding between sounds and hands that facilitates response selection through retrieval whenever both features repeat. There was no clear pattern across experiments in the movement time. Further, both experiments provided some evidence for binding and retrieval of discrete response features, which could, however, (partially) reflect a trade-off between initiation and movement time.

## General discussion

In this experimental series, we set out to investigate binding and retrieval for continuous response features, disentangling features related to the goal and the implementation of this goal. Participants therefore indicated the position of a shortly presented target circle via a swipe and a lift of their finger. We scrutinized whether an irrelevant stimulus feature, namely a sound playing with the presentation of the target, would bind and retrieve the target or the response position. Two experiments did not provide robust evidence for either of these bindings. However, the data of both experiments support binding and retrieval between a discrete relevant response feature, that is, the hand used, and both continuous response features. Hand repetitions biased responding toward the preceding target and response positions.

In the current design, we did not account for the laterality of relative response sides: Hands either moved toward an ipsilateral target (e.g., left hand moves toward target left of the vertical midline of the screen) or toward a contralateral target (e.g., left hand moves toward target right of the vertical midline of the screen).^7^ The sequence of these relative response sides was not orthogonal to target position sequence and hand sequence. For target position repetitions and hand repetitions, the relative response side always repeated (i.e., two ipsilateral or two contralateral targets). For target position repetitions and hand changes, the relative response side always changed (i.e., an ipsilateral target in trial n-1 and a contralateral target in trial n or vice versa). For target position changes, however, both hand repetitions and hand changes could produce relative response side repetitions as well as changes, depending on whether the targets in trial n and n-1 appeared on the same side of the screen. Critically, the interaction between hand sequence and target position sequences might therefore be driven by the special constellation of relative response side sequence in target position repetitions. To control for this confound, we conducted exploratory analyses by selecting only sequences with target position changes where the two targets appeared on the same side of the screen, but at different positions. For this selection, target position repetitions and changes with hand repetitions featured the same sequence of relative response side (i.e., two ipsilateral or two contralateral targets). Analogously, target position repetitions and changes with hand changes featured the same sequence of relative response side (i.e., an ipsilateral target in trial n-1 and a contralateral target in trial n or vice versa). The laterality of relative response side for this selection of trials still differed between hand repetitions and changes, but crucially, this difference was the same for both target position sequences. Importantly, in line with our original analyses, the reduction of the response error, distance, and direction change through hand repetitions was still consistently larger for target position repetitions than changes in both experiments (see *Supplementary Material* for a full report of the re-analyses of all dependent variables). The effect sizes were smaller, which is not surprising because with the exclusion of target change trials where the targets appeared on different sides of the screen (1) target position changes included changes of smaller magnitude and (2) variance was often higher because of fewer observations per participant × cell mean. Considering these re-analyses, we abide by our conclusion that hand repetitions retrieve previously bound target and response positions.

The difference in relevance of the sounds and the hand might explain why binding and retrieval did not emerge for the former but only for the latter feature. Recent evidence corroborates this assumption as the data suggested binding of response durations to relevant but not to irrelevant stimuli (Bogon et al., 2023; Pfister et al., 2022; but see Foerster et al., 2022, 2023). In the current study, participants had to follow instructions on whether to use the left or right hand, while they could, in principle, completely ignore the sounds played during target presentation as they did not matter for task completion. Still, many studies support the assumption of binding between discrete irrelevant stimulus features and discrete relevant response features although the sequential effects in response times and error rates are smaller than for relevant stimulus features (e.g., Frings et al., 2007; Giesen et al., 2012; Moeller & Frings, 2014). In line with this, if sound and hand repetitions coincided, initiation times were consistently faster in the current task (see *Supplementary Material*). This interaction pattern between sound and hand sequence suggests not only that sounds were attended to, but also that sound repetitions retrieved bindings with discrete response features.

Alternatively, irrelevant sound features and relevant response features might influence different phases of responding that do or do not coincide with the specification of continuous response features (see also He & Pratt, 2025, for an investigation of the binding time window for discrete stimulus and response features). While the relevance of continuous response features seems to increase with response execution, discrete features mainly concern response planning (e.g., Glover, 2004; Thomaschke et al., 2012). A study showed that irrelevant stimulus features, namely the words “small” and “large” affected grip aperture more strongly in the beginning of a grasping movement than in the end when the grasp got closer to the target (Glover & Dixon, 2002). The authors concluded that only response planning but not the control of the response during execution was affected by the words. Although sound sequence exerted an impact on movement times in our design when sounds had already stopped playing (see *Supplementary Material*), this impact could still stem from planning processes rather than only from the execution of the response (Tonn et al., 2023, 2025). In contrast, the hand was a relevant feature throughout the whole response, potentially being able to enter or retrieve bindings with coinciding response features at any given point in time.

The sound and the target position were perfectly contiguous and still we did not find evidence for binding and retrieval of these features. Although we outlined in the introduction that discrete response features already enter bindings as part of response plans (e.g., Mocke et al., 2022; Stoet & Hommel, 1999; Wiediger & Fournier, 2008), continuous response features might only be subject to binding or affected by retrieval after a rough response plan with discrete features has been established. This response plan might have involved sound, hand, and a general direction of the movement (e.g., straight upwards), reflected in an interaction of sound and hand sequence in initiation time (see *Supplementary Material*). Only then the actual *movement* of the left or right hand might have retrieved the preceding target position if the same hand moved. Binding between the current hand and the target position available in working memory might have happened at a similar point in time. A possible approach to test these speculations could be the investigation of (planned) overt swiping movements in an ABBA design. Only if continuous features of a goal are part of a response plan before its initiation, a hand repetition between task A and task B should retrieve the target position and task B responses should be biased toward this position relative to hand changes.

For the response position, retrieval from the hand might happen during the movement as discussed above for the target position. The feature itself might only become available when experienced at the very end of response execution and binding would then take place. At this moment, the sound had stopped playing for quite some time, but the hand was still relevant and moving. Alternatively, the response position might be estimated before reaching the final position and this estimated position might already be bound to the hand. One could argue that these estimates are informed through ongoing movement and that such (covert) anticipation triggers essentially the same processes as (overt) experience (James, 1890). It is hard to disentangle whether the implemented response features are completely based on experiences or also on estimates.

A preceding study already demonstrates that continuous features that only become available during response execution can still enter bindings. When participants had to grasp and lift objects with different uneven mass distributions from varying positions, participants placed their index and thumb more suitably for controlled object manipulation when these object features repeated than when at least one of them changed (Beyvers et al., 2022). Both features of the object were relevant for the task. While the object position was obvious already during response planning, participants could not perceive the mass distribution until they had already grasped and lifted the object. The current study demonstrates that continuous, intended aspects of a response (the target position) as well as continuous deviations from this goal (the response position) can both be bound and retrieved from the same relevant, discrete response feature (the swiping hand). For discrete responses with a dichotomous categorization of correct and erroneous responses, the evidence so far points to binding and retrieval of either response feature but not both in case of errors. If agents execute an erroneous response, relevant and irrelevant stimuli are bound to and retrieve the not executed correct response (Foerster et al., 2021, 2022, 2023) or the executed erroneous response (Foerster et al., 2026). The erroneous response also enters a binding with the effect it triggers (Foerster et al., 2022, 2026). The two-choice design in most of these studies could not assess binding and retrieval for both the correct and erroneous response at the same time, but it would only reveal the strongest of the two bindings. However, paradigms with three responses suggest binding and retrieval only between irrelevant stimuli/effects and either the correct or the erroneous response but not both (Foerster et al., 2026; Parmar et al., 2022). Taken together, bindings of categorically distinct correct or erroneous responses might feed into response planning, the execution of extended movements might instead benefit from a conciliation of preceding goals and experiences. However, more research is needed to corroborate this distinction. For example, there still is a lack of data on binding between continuous response features and effects.

Contrary to the idea of continuous features only becoming available during execution, recent electrophysiological work points at integrated feature representations for continuous features such as object shape and size for grasping movements already in the early phases of movement preparation (Guo & Niemeier, 2024). Moreover, such integrated representations seem to be modulated by current task demands (Lee et al., 2025). Because the present task was fairly simple in that participants were only required to point to a specific target location, stronger evidence for binding and retrieval of continuous response features might require paradigms with movements that aim at semantically meaningful object manipulations.

Real-world object manipulations also come with many degrees of potentially suboptimal motor interactions. Here we observed the first evidence for bindings between continuous response features that are to a certain extent erroneous, that is, the response position, and that are correct, that is, the swiping hand. As such, there can be bindings between correct and erroneous aspects of the same continuous response. An open question is whether responses that are categorized as erroneous can be bound to surrounding correct responses as has been observed for sequences of correct responses (e.g., Moeller and Frings 2019a, b). The erroneous response might bind to the not executed, correct response (Nemeth et al., 2024) or to an executed correction response which agents usually deliver quickly after erring if they get the chance (e.g., Fiehler et al., 2004, 2005; Rabbitt, 1966, 2002). Further, the erroneous response might be bound to preceding or subsequent responses. The investigation of these bindings will further specify the structure of the erroneous episode itself but also how it is embedded in a larger sequence of actions.

## Conclusion

The current study corroborates and extends the relevance of binding and retrieval for action control based on recent events. The data support the existence of bindings between response features of overt movements, among them continuous specifications of the goal and its implementation. In stark contrast to what we know from discrete response features, these continuous response features did not enter bindings with discrete, irrelevant stimulus features. This finding restricts the generalizability of principles of binding and retrieval in action control. Future investigations should explore whether such bindings emerge for other temporal feature constellations. A further pressing issue is to pinpoint when short-cuts to continuous response features are created and retrieved, that is, during the planning or during the execution of responses.

## Supplementary Information

Below is the link to the electronic supplementary material.

Supplementary Material 1

## Data Availability

The preregistrations (Supplementary Experiment: https://osf.io/gmcqz, Experiment 1: https://osf.io/6jbvn, and Experiment 2: https://osf.io/ujqew), raw data, and analysis code are publicly available in a project repository on the Open Science Framework (https://osf.io/csptq).

## Footnotes

1 In the first experiment of this series, participants always swiped with the same hand, their dominant hand. However, no reliable binding and retrieval effects with irrelevant stimulus features emerged, which is in line with recent speculations that binding and retrieval may not emerge for such direct localization responses but only for responses with a translational response mapping (Schöpper et al., 2022). Thus, the design was adapted to include a translation from color to hand. For brevity, we report the first experiment in the *Supplementary Material*.

2 There is a typo in the preregistration, stating that we would compare *color* (correct would be sound) repetitions and changes, separately for color/finger repetitions and changes in two-tailed, paired-samples *t*-tests. The hypotheses are formulated correctly in the preregistration.

3 One participant who replaced an excluded participant accidentally received a wrong assignment. Therefore, two combinations occur less/more (± 1) frequently than the others in the analyzed sample.

4 Moving from a 2:2 mapping of colors to response hands to a 4:2 mapping arguably increases working memory load and task complexity. However, such 4:2 mappings are commonplace in the literature on binding and retrieval in action control, starting from the first demonstration of distractor-response binding (Frings et al., 2007), and binding effects reliably appear even for more complex mappings (e.g., 6:3 in Foerster et al., 2023, 2026, in press).

5 This exclusion was not specified in the preregistration.

6 We accidently copy-and-pasted a sentence stating that we would select target position repetitions into the section on outliers and exclusions in the preregistration. In the section on analyses, we correctly list target location sequence with its two levels, that is, repetitions and changes, as factor in the ANOVA. This analysis is also in line with the hypotheses in the preregistration.

7 We thank an anonymous reviewer for pointing us to this important aspect.
