# Supplementary material for: Binding continuous response features of extended movements: Integration with discrete response but not stimulus features
Source: Psychol Res. 2026 Apr 22;90(3):77. doi: 10.1007/s00426-026-02295-5 (PMC13102753; doi:10.1007/s00426-026-02295-5)
Supplement: Supplementary file 1 — Supplementary Material 1 [file 426_2026_2295_MOESM1_ESM.pdf]

Supplementary material for

Binding continuous response features of extended movements: Integration with discrete response but not stimulus features

in *Psychological Research*

Anna Foerster, Birte Moeller, Maria Nemeth, Moritz Schaaf, Christian Frings, & Roland Pfister*

Trier University, Universitätsring 15, 54296 Trier, Germany

* Correspondence to roland.pfister@uni-trier.de

Supplementary experiment

Introduction

In this supplementary experiment, participants responded with the index finger of their dominant hand, swiping it from only one starting area to the target circle (see Fig. S1). As in Experiment 1, the target always had one of two colors and was accompanied by one of two sounds. Yet, in contrast to Experiment 1 and 2, the target color was irrelevant, and its identity was perfectly correlated with the identity of the irrelevant sound. To facilitate comparison with the main experiments, where the target color was a relevant feature and the sound an irrelevant feature, the irrelevant sound/color compound is referred to as *sound*.

As in the primary analyses of Experiment 1 of the main text, we assessed binding of the target position and the response position to the irrelevant sound in sequences of successive trials with the same target position. We had the same hypotheses, that is, sound repetition benefits would deliver empirical support for binding and retrieval of the target position if they are evident in the response error and for binding and retrieval of the response position if they are evident in the response distance and direction change. We also assessed whether sound repetition benefits would manifest in the initiation time and the movement time. While such benefits would point to binding and retrieval in action planning and execution, these benefits do not specify the bound response features. Finally, we assessed whether the magnitude of the response error during binding (i.e., in the previous trial n-1) would relate to larger sound repetition benefits in the response error and distance.

Method

Participants

For categorical, erroneous responses, previous binding and retrieval effects between irrelevant stimulus features and goal-related response features were $d_z = 0.40$ in response times (Foerster et al., 2021). At $\alpha = 5\%$, a sample of 40 participants has a power of $1 - \beta \geq 80\%$ to detect this effect size in a one-tailed *t*-test (computed with the *power.t.test* function in *R* version 4.2.2; R Core Team, 2023).

As in the main experiments, we planned to invite pilot samples of eight participants each. Our goal was to exclude a maximum of 25% of participants from such a pilot sample (see *Data treatment*) and to adapt the study design and invite a new pilot sample if we did not meet this goal. However, our first study met this goal, so we filled up the sample according to the power analysis above.

Forty-four participants conducted our experiment because we had to exclude the datasets from four participants (see *Data treatment*). All participants provided informed consent. The 40 participants who entered our analysis had a mean age of 29.6 years (SD = 11.8 years). Thirty-one participants identified as female, nine as male, nobody as non-binary. Six participants reported to be left-handed, 34 to be right-handed and nobody to be ambidextrous.

Apparatus, stimuli, and procedure

The apparatus, stimuli, and procedure of this experiment were like in Experiment 1 in the main text, but with the following changes:

The experiment was conducted on slightly older tablets (iPad Pro, 3rd generation) in portrait mode. The application had a resolution of 1366 × 1024 pixels and was upscaled to fill the whole 12.9-inch screen. As in Experiment 1, the target always had one of two colors and was accompanied by one of two sounds (see Fig. S1). However, contrary to Experiment 1, color did not indicate which hand had to be used in this experiment. Rather, participants always used the same hand for their swiping movements. Consequently, there was only one starting area, and the side of the starting area was dependent on the self-reported hand preference of the participants. Thus, both colors and sounds were irrelevant. In the current experiment, colors and sounds were perfectly correlated, and the assignment of colors to sounds was counterbalanced across participants.

Figure S1

Sequential trial structure

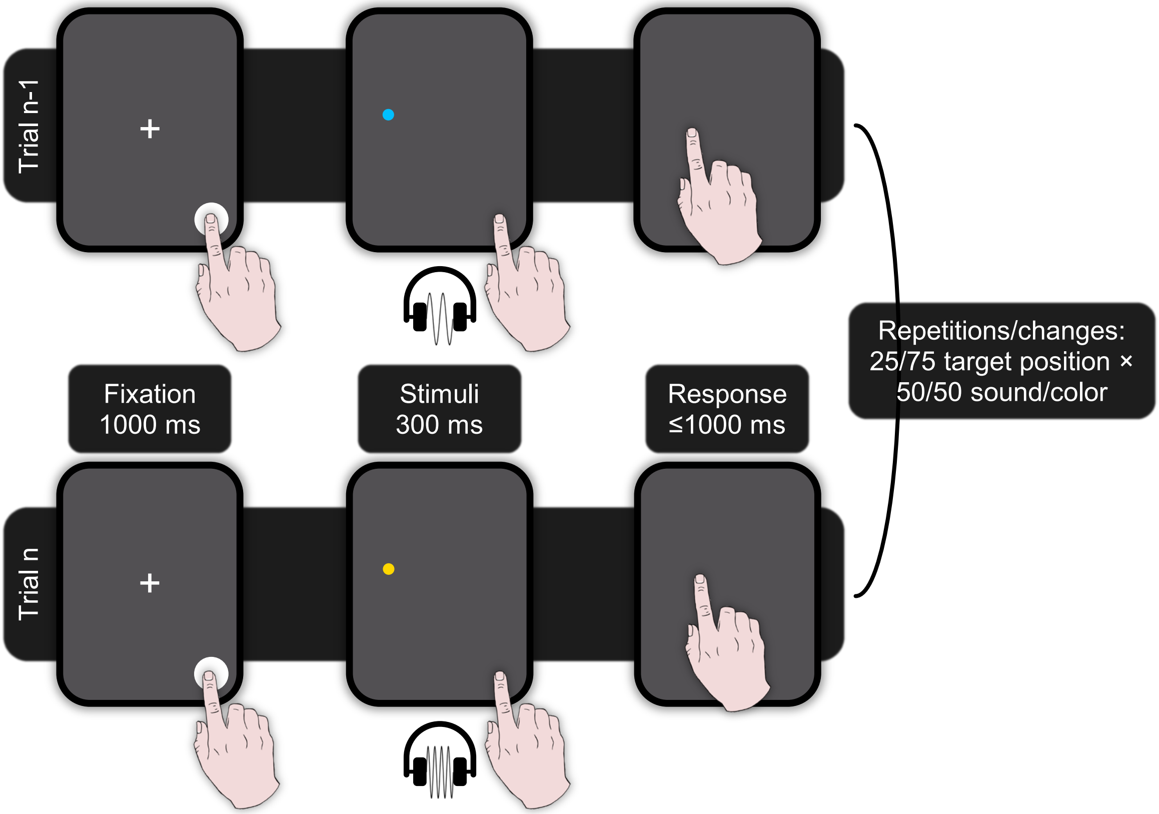

Note. The starting area appeared at the left or right side of the screen, depending on the participants' dominant hand. Participants had to touch the starting area during the presentation of the fixation cross. After 1000 ms, a target circle appeared for 300 ms, accompanied by one of two sounds. The color of the target and the sound were perfectly correlated and both irrelevant. After the target onset, they had 1000 ms to swipe as closely to the position of the (already vanished) target as possible and lift their finger. In successive trials (trial n-1 and n), the target position repeated in 25% of the trials and the color and the sound repeated in 50% of the trials.

The experiment had thirteen blocks. The first block was considered practice and had 25 trials. The remaining twelve blocks had 57 experimental trials each. In the first trial of each block, the position of the target and the color/sound compound were chosen randomly. In the remaining trials of the block, the target position was random in 75% of the trials (target position change). In the other 25% of the trials, the target position was the same as in the preceding trial (target position repetition). The sound compound repeated its identity in successive trials in half of the trials and changed its identity in the other half. Therefore, there were four possible combinations of target position and sound sequence. The two sequence combinations with target position repetitions occurred seven times in each experimental block (and three times in the practice block). The two sequence combinations with target position changes occurred 21 times in each experimental block (and nine times in the practice block).

The fixation was shorter than in the other two experiments, it lasted 1000 ms (rather than 1250 ms), and participants had to respond faster, that is, within 1000 ms after target onset (rather than 1250 ms).

Because only one finger was used throughout the whole experiment, there were fewer types of errors: (1) participants began a touch outside of the starting area (German message: “Start nicht getroffen!”, translation: “Start not hit!”), (2) they did not touch the starting area before target onset (German message: “Schneller zurück zum Start!”, translation: “Faster back to the start!”), (3) they swiped out of the starting area before target onset (German message: “Zu früh gestartet!”, translation: “Started too early!”), (4) they lifted their finger from the screen before target onset (German message: “Zu früh beendet!”, translation: “Ended too early!”), (5) they lifted their finger from the screen after target onset within the starting area (German message: “Bitte Finger zum Zielkreis bewegen!”, translation: “Please move finger toward target circle!”), (6) they did not lift their finger from the screen within the response deadline after leaving the starting area (German message: “Beende deine Bewegung zügiger!”, translation: “Finish your movement faster!”).

Results

Data treatment

Our exclusion criteria were the same as for Experiment 1 in the main text. One participant aborted the experiment prematurely and was excluded and replaced.[1] We excluded the first block and the first trial of each block. We excluded and replaced participants who committed more than 40% errors (two participants). For the remaining participants, we excluded erroneous trials (4.87%) and then the trials immediately following these errors (4.17%). We excluded and replaced one participant who had more than 40% trials with low precision. We then excluded trials with low precision (0.82%) for the remaining participants. We selected only trials with the same circle position as the preceding trial (25.20%). Finally, we identified outliers. At this point, we noticed that ten trials had an initiation time of 0 with no logged movement time. We excluded these trials (0.16%), deviating from our preregistration. We then excluded trials with outliers (8.26%). All participants delivered at least 10 observations in both design cells after these exclusions and were included in the analyses.

[1] This exclusion was not specified in the preregistration.

Main analyses

We compared whether our three main dependent variables, that is, response error, response distance, and response direction change were smaller for sound repetitions than changes in paired-samples one-tailed *t*-tests.

For the response error and distance, the differences were not significant (see Fig. S2A and B), $|t| < 1$. The response direction change was smaller for sound repetitions than changes (see Fig. S2C), $t(39) = 1.91$, $p = .032$, $d_z = 0.30$.

Figure S2

Main results of the supplementary experiment

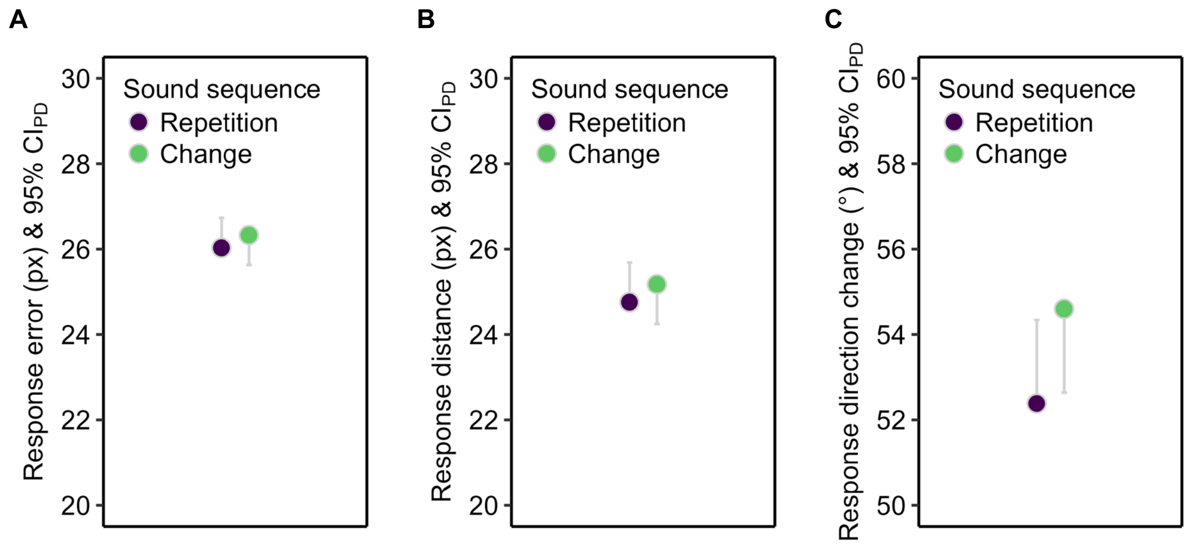

Note. Means for sound repetitions (purple) and changes (green) for **A)** the response error in pixels (px), **B)** the response distance in px, and **C)** the response direction change in degrees (°). The error bars are 95% confidence intervals of the paired differences (CI_{PD}), visualizing a one-tailed *t*-test between sound sequences.

Secondary analyses

We conducted the same tests for the initiation time and the movement time. Although these temporal variables were descriptively shorter for sound repetitions than changes (see Fig. S3), these differences were not significant in both the initiation time, $t(39) = 1.68$, $p = .051$, $d_z = 0.27$, and the movement time, $t(39) = 1.36$, $p = .092$, $d_z = 0.21$.

Figure S3

Results for the temporal variables of the supplementary experiment

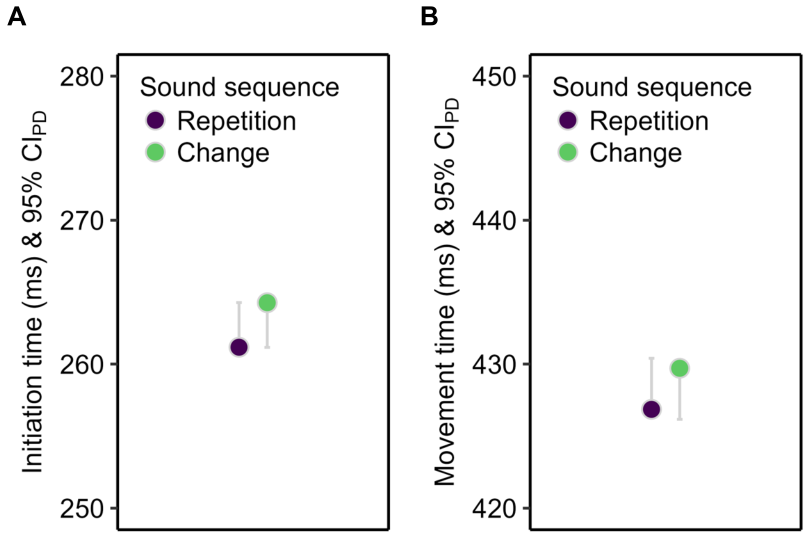

Note. Means for sound repetitions (purple) and changes (green) for **A)** the initiation time, and **B)** the movement time in milliseconds (ms). The error bars are 95% confidence intervals of the paired differences (CI_{PD}), visualizing a one-tailed *t*-test between sound sequences.

Finally, we assessed whether the previous response error correlated positively with the sound sequence effect ($\Delta \coloneqq$ sound change – sound repetition) in the response error and the response distance. Both correlations were not significant, $|r|$s ≤ .20, *p*s ≥ .111.

Exploratory data analyses

The exploration of the continuously logged touch data revealed that one participant initiated multiple touches in 41.3% of their analyzed trials. We could not replicate this behavior in the program. We repeated all analyses without the data of this participant. The statistical decisions did not change for the response error, $t(38) = 1.41$, $p = .083$, $d_z = 0.23$, the response distance, $|t| < 1$, the initiation time, $t(38) = 1.56$, $p = .064$, $d_z = 0.25$, and the movement time, $t(38) = 1.19$, $p = .122$, $d_z = 0.19$, or for the correlations, $|r|$s ≤ .20, *p*s ≥ .112. However, after this exclusion, the comparison for the response direction change was not significant anymore, $t(38) = 1.63$, $p = .055$, $d_z = 0.26$.

Note that the same exploration of the continuously logged touch data was also conducted for Experiments 1 and 2. However, in these experiments, close to none of the trials were multi-touch swipes, and consequently, the data pattern was not affected in these experiments.

Discussion

Although sound repetitions had descriptively lower values in all five dependent measures than sound changes, this benefit was only significant for response direction change. However, even for the response direction change, this benefit was not significant anymore when we excluded a dataset with unexpected touch patterns. Therefore, this experiment does not deliver convincing support for binding between stimulus features and continuous response features related to the goal or its implementation. There was also no indication of binding and retrieval in the initiation time and the movement time.

However, this data pattern resonates with a recent study that indicates that binding and retrieval in localization tasks may only emerge if a translational step is involved in response selection (Schöpper et al., 2022; see also Wiediger & Fournier, 2008). Binding and retrieval effects were absent when participants touched a target area directly, but they were present when participants had to touch an area opposite to the presented target. In our current experiment, participants also swiped directly from the starting area to a target. In the two experiments that we present in the main text, we included a translation by instructing participants via the target color in each trial whether to use their left or right index finger to indicate the target position.

Experiment 1

Secondary analyses

For Experiment 1, we first conducted the same analyses as for the main dependent measures for the initiation time and the movement time. The initiation time was shorter for hand repetitions compared to changes (see Fig. S4), $F(1, 51) = 230.87$, $p < .001$, $\eta_p^2 = .82$, and for sound repetitions compared to changes, $F(1, 51) = 12.67$, $p = .001$, $\eta_p^2 = .20$. The interaction of the two factors was significant, $F(1, 51) = 10.55$, $p = .002$, $\eta_p^2 = .17$, indicating that sound repetitions were faster than sound changes for hand repetitions, $t(51) = 4.79$, $p < .001$, $d_z = 0.66$, but not for changes, $|t| < 1$.

Figure S4

Descriptive statistics for the initiation time in Experiment 1

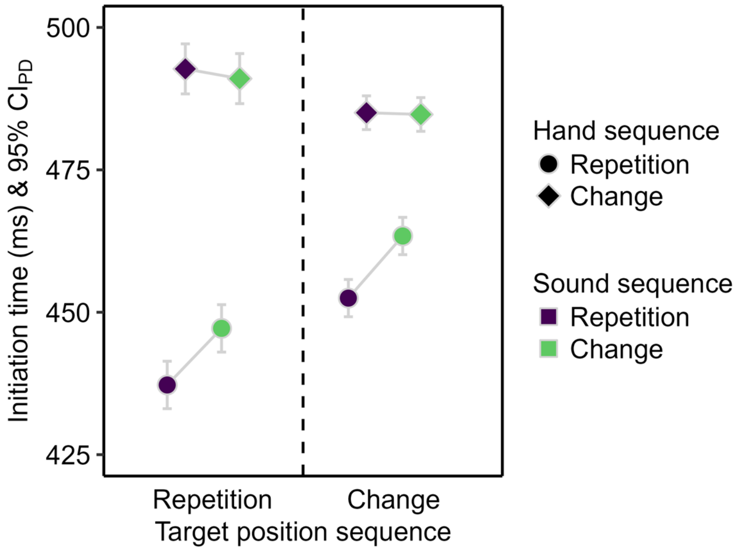

Note. Mean initiation time in milliseconds (ms). Means are depicted as a function of sound repetition (purple) and change (green), hand repetition (circle) and change (diamond), and target position repetition (left) and change (right). The error bars are 95% confidence intervals of paired differences (CI_{PD}), visualizing two-tailed *t*-tests between sound sequences.

The movement time was longer for hand repetitions than changes (see Fig. S5), $F(1, 51) = 8.18$, $p = .006$, $\eta_p^2 = .14$. The main effect of sound sequence was not significant, $F < 1$. The two-way interaction was significant, $F(1, 51) = 4.26$, $p = .044$, $\eta_p^2 = .08$, indicating that sound repetitions were descriptively faster than changes for hand repetitions, $t(51) = 1.25$, $p = .218$, $d_z = 0.17$, while sound repetitions were descriptively slower than changes for hand changes, $t(51) = -1.68$, $p = .098$, $d_z = -0.23$.

Figure S5

Descriptive statistics for the movement time in Experiment 1

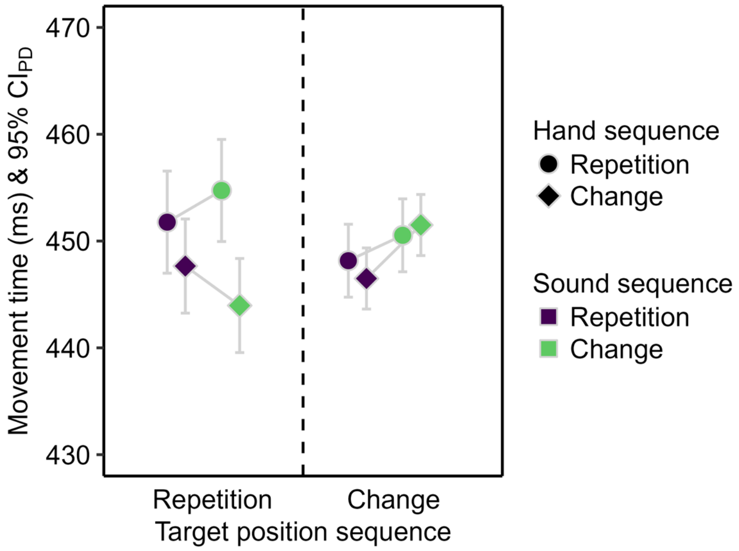

Note. Mean movement time in milliseconds (ms). Means are depicted as a function of sound repetition (purple) and change (green), hand repetition (circle) and change (diamond), and target position repetition (left) and change (right). The error bars are 95% confidence intervals of paired differences (CI_{PD}), visualizing two-tailed *t*-tests between sound sequences.

Second, we investigated whether binding and retrieval effects are modulated by the size of the response error in the previous trial. Therefore, we computed the median of the response error in trial n-1 separately for each combination of hand and sound sequence and participant. We then split the data into trials having lower or equal previous response errors versus trials having higher previous response errors than the corresponding cell median. All five dependent variables were subsequently analyzed with 2 × 2 × 2 ANOVAs with the within-subjects factors previous response error (small vs. high) × hand sequence (repetition vs. change) × sound sequence (repetition vs. change). To avoid redundancy with the primary analyses in the main manuscript, we report only main effects of response error in trial n-1 as well as interactions with this factor. Descriptive statistics are summarized in Table S1.

Table S1

Descriptive statistics for the impact of the previous response error

Dependent variable	Previous response error	Hand sequence	Sound sequence	Mean	Standard deviation
Response error	Small	Repetition	Repetition	26.7	13.6
			Change	27.7	14.5
		Change	Repetition	32.9	18.0
			Change	33.5	16.7
	High	Repetition	Repetition	39.0	23.3
			Change	38.2	25.0
		Change	Repetition	37.5	21.7
			Change	38.7	22.4
Response distance	Small	Repetition	Repetition	22.2	7.1
			Change	22.0	7.0
		Change	Repetition	37.5	21.2
			Change	36.1	18.5
	High	Repetition	Repetition	31.6	13.2
			Change	31.6	12.3
		Change	Repetition	56.4	31.7
			Change	55.6	31.6
Response direction change	Small	Repetition	Repetition	57.4	19.7
			Change	53.4	20.3
		Change	Repetition	83.2	17.3
			Change	78.3	16.9
	High	Repetition	Repetition	37.7	17.9
			Change	37.3	16.7
		Change	Repetition	75.4	17.2
			Change	73.5	19.1
Initiation time	Small	Repetition	Repetition	436.0	34.7
			Change	443.8	32.4
		Change	Repetition	491.5	42.5
			Change	490.4	40.2
	High	Repetition	Repetition	438.4	28.8
			Change	450.8	31.4
		Change	Repetition	494.0	40.9
			Change	491.6	44.9
Movement time	Small	Repetition	Repetition	458.9	87.5
			Change	462.1	89.3
		Change	Repetition	456.9	79.2
			Change	453.7	76.0
	High	Repetition	Repetition	444.4	87.9
			Change	446.9	88.8
		Change	Repetition	438.2	79.0
			Change	433.5	78.9

The response error was smaller when the previous response error was small rather than high, $F(1, 51) = 44.80$, $p < .001$, $\eta_p^2 = .47$. The interaction of previous response error and hand sequence was significant, $F(1, 51) = 36.52$, $p < .001$, $\eta_p^2 = .42$, because hand repetitions reduced the response error when the previous response error was small, $t(51) = 7.09$, $p < .001$, $d_z = 0.98$, but not when it was high, $t(51) = -0.63$, $p = .534$, $d_z = -0.09$. The other two-way interaction and the three-way interaction were not significant, $F \leq 2.07$, $p \geq .156$, $\eta_p^2 \leq .04$.

The response distance was smaller when the previous response error was small rather than high, $F(1, 51) = 129.69$, $p < .001$, $\eta_p^2 = .72$. The interaction of previous response error and hand sequence was significant, $F(1, 51) = 25.26$, $p < .001$, $\eta_p^2 = .33$, because hand repetitions reduced the response distance more strongly when the previous response error was small, $t(51) = 6.77$, $p < .001$, $d_z = 0.94$, than when it was high, $t(51) = 6.52$, $p < .001$, $d_z = 0.90$. The other two-way interaction and the three-way interaction were not significant, $F < 1$.

The response direction change was larger when the previous response error was small rather than high, $F(1, 51) = 81.89$, $p < .001$, $\eta_p^2 = .62$. The interaction of previous response error and hand sequence was significant, $F(1, 51) = 32.21$, $p < .001$, $\eta_p^2 = .39$, because hand repetitions reduced the response direction change less strongly when the previous response error was small, $t(51) = 9.68$, $p < .001$, $d_z = 1.34$, than when it was high, $t(51) = 13.50$, $p < .001$, $d_z = 1.87$. The other two-way interaction and the three-way interaction were not significant, $F \leq 2.61$, $p \geq .112$, $\eta_p^2 \leq .05$.

The initiation time was shorter when the previous response error was small rather than high, $F(1, 51) = 4.73$, $p = .034$, $\eta_p^2 = .08$. Both two-way interactions and the three-way interaction were not significant, $F \leq 1.52$, $p \geq .223$, $\eta_p^2 \leq .03$.

The MT was longer when the previous response error was small rather than high, $F(1, 51) = 28.51$, $p < .001$, $\eta_p^2 = .36$. Both two-way interactions and the three-way interaction were not significant, $F \leq 1.74$, $p \geq .193$, $\eta_p^2 \leq .03$.

Exploratory re-analyses

First, we analyzed the initiation time and the movement time in 2 × 2 × 2 ANOVAs with the within-subjects factors target position sequence (repetition vs. change) × hand sequence (repetition vs. change) × sound sequence (repetition vs. change). In case of significant three-way interactions, we conducted a 2 × 2 ANOVA only for target position changes (for results on target position repetitions, see

Secondary analyses in *Experiment 1*). Significant two-way interactions were followed by separate two-tailed, paired-samples *t*-tests.

The initiation time was shorter for target position repetitions than changes (see Fig. S4), $F(1, 51) = 23.35$, $p < .001$, $\eta_p^2 = .31$, for hand repetitions than changes, $F(1, 51) = 160.85$, $p < .001$, $\eta_p^2 = .76$, and for sound repetitions than changes, $F(1, 51) = 30.51$, $p < .001$, $\eta_p^2 = .37$. The interaction of target position and hand sequence was significant, $F(1, 51) = 115.76$, $p < .001$, $\eta_p^2 = .69$, indicating a larger benefit of hand repetitions compared to changes for target position repetitions, $t(51) = 15.19$, $p < .001$, $d_z = 2.11$, than changes, $t(51) = 8.61$, $p < .001$, $d_z = 1.19$. The two-way interaction of hand and sound sequence was also significant, $F(1, 51) = 25.03$, $p < .001$, $\eta_p^2 = .33$, because there was a significant benefit for sound repetitions relative to changes for hand repetitions, $t(51) = 7.61$, $p < .001$, $d_z = 1.06$, but not for changes, $|t| < 1$. The remaining two-way and three-way interactions were not significant, $F < 1$.

In the movement time, the main effect of target position sequence was not significant (see Fig. S5), $F < 1$. The movement time was longer for hand repetitions than changes, $F(1, 51) = 4.11$, $p = .048$, $\eta_p^2 = .07$. The main effect of sound sequence was not significant, $F(1, 51) = 2.57$, $p = .115$, $\eta_p^2 = .05$. The two-way interaction of target position and hand sequence was significant, $F(1, 51) = 11.31$, $p = .001$, $\eta_p^2 = .18$, because there were significant costs for hand repetitions relative to changes for target position repetitions, $t(51) = -2.86$, $p = .006$, $d_z = -0.40$, but not for changes, $|t| < 1$. The two-way interaction of target position and sound sequence was also significant, $F(1, 51) = 4.84$, $p = .032$, $\eta_p^2 = .09$, because there was no effect of sound sequence for target position repetitions, $|t| < 1$, but a benefit for sound repetitions over changes for target position changes, $t(51) = 3.35$, $p = .002$, $d_z = 0.47$. The factors hand and sound sequence did not interact significantly, $F(1, 51) = 1.02$, $p = .317$, $\eta_p^2 = .02$. The three-way interaction of all factors was significant, $F(1, 51) = 5.81$, $p = .020$, $\eta_p^2 = .10$. The results for target position repetitions are reported above (see *Secondary analyses*). For target position changes, only the main effect of sound sequence was significant, $F(1, 51) = 11.25$, $p = .002$, $\eta_p^2 = .18$, while the main effect of hand sequence, $F < 1$, and the two-way interaction, $F(1, 51) = 1.38$, $p = .245$, $\eta_p^2 = .03$, were not significant.

Exploratory re-analyses of a selected dataset

Second, we repeated the analyses described above for each dependent variable, this time, for a selected dataset where we controlled for the laterality of response side. We selected target position

changes where the two targets appeared on the same side of the screen but at different positions for hand repetitions (48.5% trials excluded) and hand changes (50.3% trials excluded). Therefore, target position repetitions and changes with hand repetitions featured the same sequence of response directions (i.e., two ipsilateral or two contralateral targets). Analogously, target position repetitions and changes with hand changes featured the same sequence of response directions (i.e., an ipsilateral target in trial n-1 and a contralateral target in trial n or vice versa). All participants still contributed at least 10 observations in each design cell.

The response error was smaller for target position repetitions than changes (see Fig. S6), $F(1, 51) = 4.15$, $p = .047$, $\eta_p^2 = .08$, and for hand repetitions than changes, $F(1, 51) = 22.62$, $p < .001$, $\eta_p^2 = .31$. The main effect of sound sequence was not significant, $F < 1$. The two-way interaction between target position and hand sequence was significant, $F(1, 51) = 5.02$, $p = .029$, $\eta_p^2 = .09$, because the reduction of the response error for hand repetitions compared to changes was larger for target position repetitions, $t(51) = 4.27$, $p < .001$, $d_z = 0.59$, than changes, $t(51) = 3.60$, $p = .001$, $d_z = 0.50$. The two-way interaction between target position and sound sequence was also significant, $F(1, 51) = 8.24$, $p = .006$, $\eta_p^2 = .14$, because sound repetitions descriptively reduced the response error compared to sound changes when the target position repeated, $t(51) = 1.23$, $p = .223$, $d_z = 0.17$, but significantly increased the response error when the target position changed, $t(51) = -2.14$, $p = .037$, $d_z = -0.30$. The remaining two-way interaction between hand and sound sequence and the three-way interaction were not significant, $F < 1$.

Figure S6

Descriptive statistics for the response error in Experiment 1

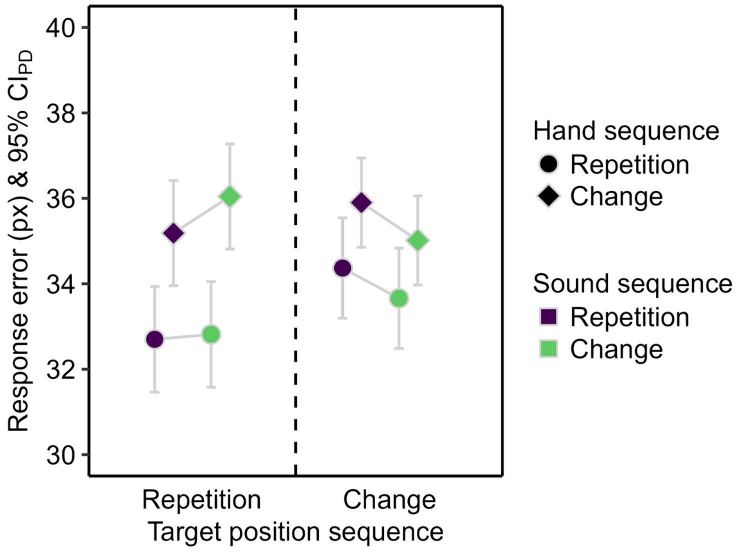

Note. Mean response error in pixels (px). Means are depicted as a function of sound repetition (purple) and change (green), hand repetition (circle) and change (diamond), and target position repetition (left) and change (right). The error bars are 95% confidence intervals of paired differences (CI_{PD}), visualizing two-tailed *t*-tests between sound sequences.

The response distance was shorter for target position repetitions than changes (see Fig. S7), $F(1, 51) = 14983.68$, $p < .001$, $\eta_p^2 > .99$, and for hand repetitions than changes, $F(1, 51) = 26.42$, $p < .001$, $\eta_p^2 = .34$. The main effect of sound sequence was not significant, $F < 1$. The two-way interaction between target position and hand sequence was significant, $F(1, 51) = 9.95$, $p = .003$, $\eta_p^2 = .16$, because the reduction of the response distance for hand repetitions compared to changes was larger for target position repetitions, $t(51) = 6.69$, $p < .001$, $d_z = 0.93$, than changes, $t(51) = 2.21$, $p = .031$, $d_z = 0.31$. The remaining two-way interactions, $F \leq 2.62$, $p \geq .112$, $\eta_p^2 \leq .05$, and the three-way interaction were not significant, $F(1, 51) = 3.65$, $p = .062$, $\eta_p^2 = .07$.

Figure S7

Descriptive statistics for the response distance in Experiment 1

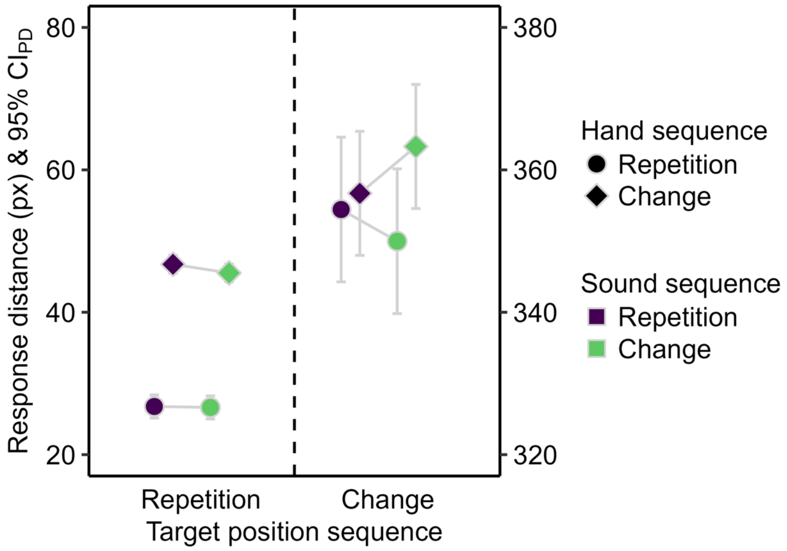

Note. Mean response distance in pixels (px). Means are depicted as a function of sound repetition (purple) and change (green), hand repetition (circle) and change (diamond), and target position repetition (left) and change (right). The error bars are 95% confidence intervals of paired differences (CI_{PD}), visualizing two-tailed *t*-tests between sound sequences. Changing the target position inevitably resulted in longer response distances than repeating the target position. Therefore, target position sequences are mapped to separate y-axes to facilitate the comparison of sound and hand sequence effects.

The response direction change was smaller for target position repetitions than changes (see Fig. S8), $F(1, 51) = 10.79$, $p = .002$, $\eta_p^2 = .17$, and for hand repetitions than changes, $F(1, 51) = 122.41$, $p < .001$, $\eta_p^2 = .71$. The main effect of sound sequence was not significant, $F(1, 51) = 3.83$, $p = .056$, $\eta_p^2 = .07$. The interaction of target position and hand sequence was significant, $F(1, 51) = 46.10$, $p < .001$, $\eta_p^2 = .47$, because the reduction of the response direction change for hand repetitions compared to changes was larger for target position repetitions, $t(51) = 12.58$, $p < .001$, $d_z = 1.75$, than changes, $t(51) = 8.64$, $p < .001$, $d_z = 1.20$. The interaction of target position and sound sequence was also significant, $F(1, 51) = 4.27$, $p = .044$, $\eta_p^2 = .08$, because sound repetitions significantly increased the response direction change compared to sound changes when the target position repeated, $t(51) = -2.74$, p = .008, d = -0.38, but did not significantly modulate response direction change when the target position changed, $|t| < 1$. The two-way interaction between hand and sound sequence, $F < 1$, and the three-way interaction, $F(1, 51) = 1.46$, $p = .233$, $\eta_p^2 = .03$, were not significant.

Figure S8

Descriptive statistics for the response direction change in Experiment 1

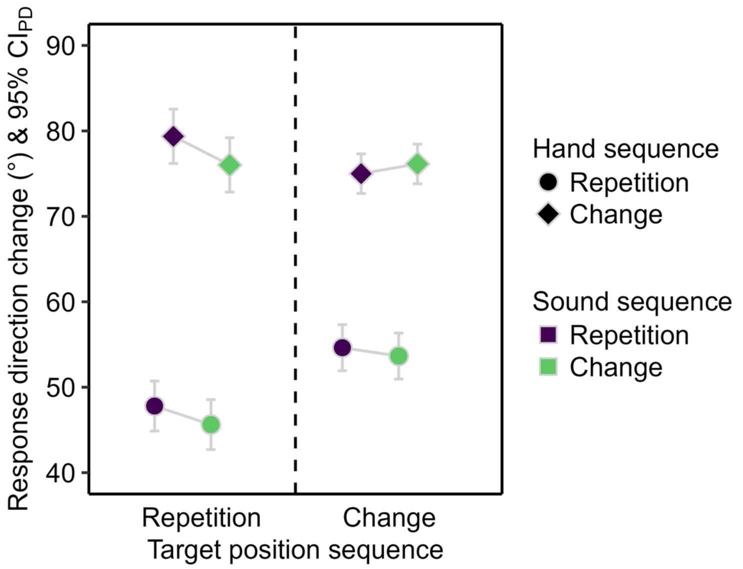

Note. Mean (absolute) response direction change in degrees (°). Means are depicted as a function of sound repetition (purple) and change (green), hand repetition (circle) and change (diamond), and target position repetition (left) and change (right). The error bars are 95% confidence intervals of paired differences (CI_{PD}), visualizing two-tailed *t*-tests between sound sequences.

The initiation time was shorter for target position repetitions than changes (see Fig. S9), $F(1, 51) = 19.40$, $p < .001$, $\eta_p^2 = .28$, for hand repetitions than changes, $F(1, 51) = 186.96$, $p < .001$, $\eta_p^2 = .79$, and for sound repetitions than changes, $F(1, 51) = 22.24$, $p < .001$, $\eta_p^2 = .30$. The interaction of target position and hand sequence was significant, $F(1, 51) = 57.41$, $p < .001$, $\eta_p^2 = .53$, indicating a larger benefit of hand repetitions compared to changes for target position repetitions, $t(51) = 15.19$, $p < .001$, $d_z = 2.11$, than changes, $t(51) = 10.77$, $p < .001$, $d_z = 1.49$. The two-way interaction of hand and sound sequence was also significant, $F(1, 51) = 23.08$, $p < .001$, $\eta_p^2 = .31$, because there was a significant benefit for sound repetitions relative to changes for hand repetitions, $t(51) = 6.87$, $p < .001$, $d_z = 0.95$, but not for changes, $|t| < 1$. The remaining two-way and three-way interactions were not significant, $F < 1$.

Figure S9

Descriptive statistics for the initiation time in Experiment 1

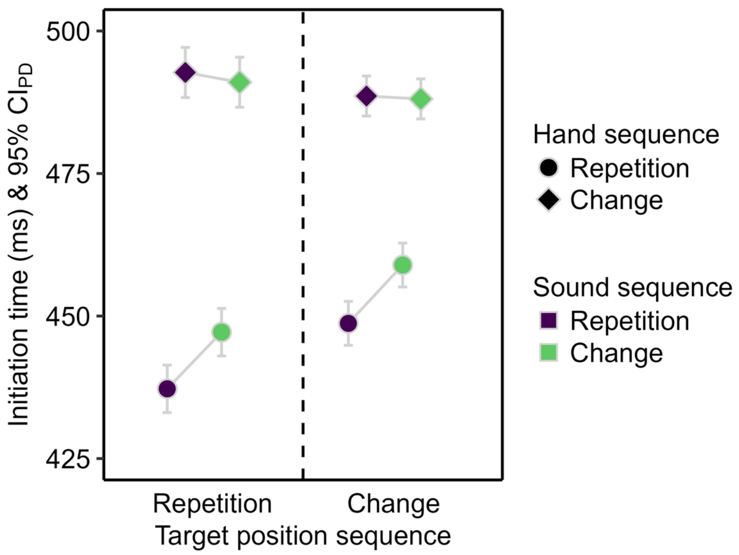

Note. Mean initiation time in milliseconds (ms). Means are depicted as a function of sound repetition (purple) and change (green), hand repetition (circle) and change (diamond), and target position repetition (left) and change (right). The error bars are 95% confidence intervals of paired differences (CI_{PD}), visualizing two-tailed *t*-tests between sound sequences.

In the movement time, the main effect of target position sequence was not significant (see Fig. S10), $F < 1$. The movement time was longer for hand repetitions than changes, $F(1, 51) = 5.02$, $p = .029$, $\eta_p^2 = .09$. The main effect of sound sequence was not significant, $F(1, 51) = 3.61$, $p = .063$, $\eta_p^2 = .07$. The two-way interaction of target position and hand sequence was significant, $F(1, 51) = 6.90$, $p = .011$, $\eta_p^2 = .12$, because there were significant costs for hand repetitions relative to changes for target position repetitions, $t(51) = -2.86$, $p = .006$, $d_z = -0.40$, but not for changes, $|t| < 1$. The two-way interaction of target position and sound sequence was also significant, $F(1, 51) = 5.80$, $p = .020$, $\eta_p^2 = .10$, because there was no effect of sound sequence for target position repetitions, $|t| < 1$, but a benefit for sound repetitions over changes for target position changes, $t(51) = 3.13$, $p = .003$, $d_z = 0.43$. The factors hand and sound sequence did not interact significantly, $F < 1$. The three-way interaction of all factors was significant, $F(1, 51) = 5.38$, $p = .024$, $\eta_p^2 = .10$. The results for the same selection of target position repetitions are reported above (see *Secondary analyses*). For target position changes, only the main effect of sound sequence was significant, $F(1, 51) = 9.78$, $p = .003$, $\eta_p^2 = .16$ while the main effect of hand sequence, $F < 1$, and the two-way interaction, $F(1, 51) = 1.78$, $p = .188$, $\eta_p^2 = .03$, were not significant.

Figure S10

Descriptive statistics for the movement time in Experiment 1

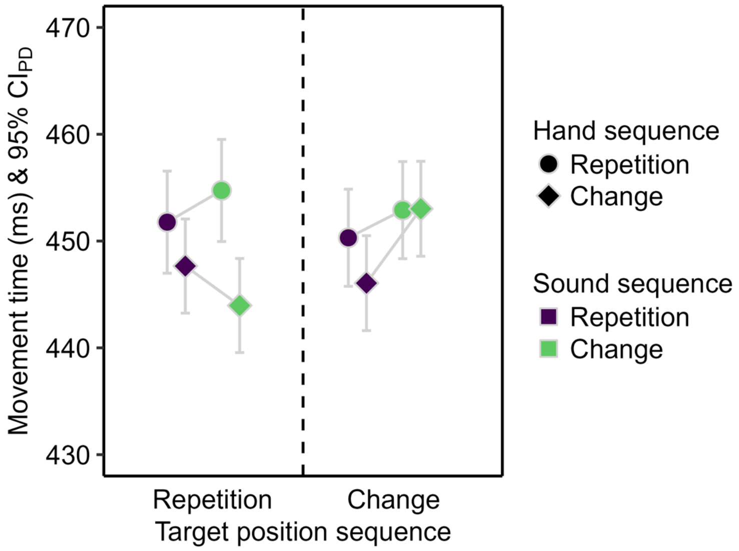

Note. Mean movement time in milliseconds (ms). Means are depicted as a function of sound repetition (purple) and change (green), hand repetition (circle) and change (diamond), and target position repetition (left) and change (right). The error bars are 95% confidence intervals of paired differences (CI_{PD}), visualizing two-tailed *t*-tests between sound sequences.

Experiment 2

Secondary analyses

We conducted the same analyses as for the main dependent measures for the initiation time and movement time. The initiation time was shorter for target position repetitions than changes (see Fig. S11), $F(1, 95) = 217.99$, $p < .001$, $\eta_p^2 = .70$, for hand repetitions than changes, $F(1, 95) = 67.04$, $p < .001$, $\eta_p^2 = .41$, and for sound repetitions than changes, $F(1, 95) = 28.29$, $p < .001$, $\eta_p^2 = .23$. The interaction of target position and hand sequence was significant, $F(1, 95) = 158.58$, $p < .001$, $\eta_p^2 = .63$, indicating a larger benefit of hand repetitions relative to changes for target position repetitions, $t(95) = 10.76$, $p < .001$, $d_z = 1.10$, than changes, $t(95) = 4.62$, $p < .001$, $d_z = 0.47$. The two-way interaction of hand and sound sequence was also significant, $F(1, 95) = 73.15$, $p < .001$, $\eta_p^2 = .44$, because there was a significant benefit for sound repetitions relative to changes for hand repetitions, $t(95) = 10.60$, $p < .001$, $d_z = 1.08$, but a descriptive cost for changes, $t(95) = -1.93$, $p = .056$, $d_z = -0.20$. The remaining two-way and three-way interactions were not significant, $F < 1$.

Figure S11

Descriptive statistics for the initiation time in Experiment 2

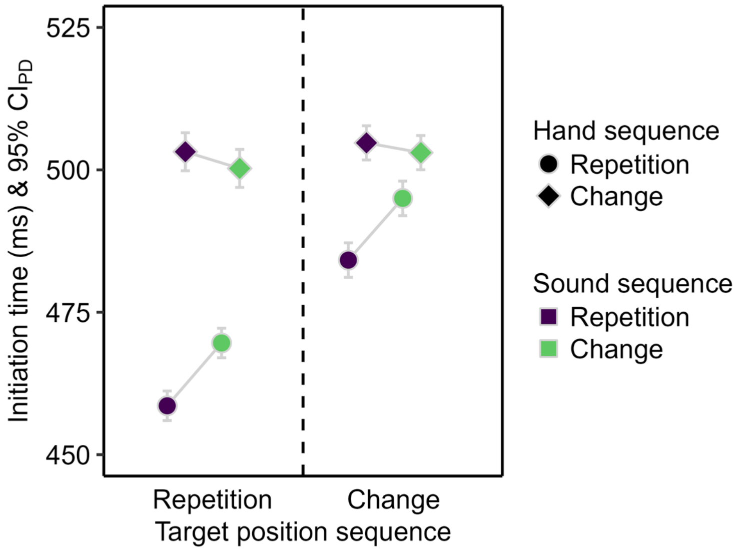

Note. Mean initiation time in milliseconds (ms). Means are depicted as a function of sound repetition (purple) and change (green), hand repetition (circle) and change (diamond), and target position repetition (left) and change (right). The error bars are 95% confidence intervals of paired differences (CI_{PD}), visualizing two-tailed *t*-tests between sound sequences.

In the movement time, the main effects of target position sequence and hand sequence were not significant (see Fig. S12), $F < 1$. The movement time was shorter for sound repetitions than changes, $F(1, 95) = 31.57$, $p < .001$, $\eta_p^2 = .25$. The two-way interaction of target position and hand sequence was

significant, $F(1, 95) = 5.45$, $p = .022$, $\eta_p^2 = .05$, because there were descriptive costs for hand repetitions relative to changes for target position repetitions, $|t| < 1$, but descriptive hand repetition benefits for target position changes, $t(95) = 1.69$, $p = .094$, $d_z = 0.17$. The remaining two-way interactions were not significant, $F \leq 2.43$, $p \geq .122$, $\eta_p^2 \leq .02$. The three-way interaction of all factors was also not significant, $F(1, 95) = 3.40$, $p = .068$, $\eta_p^2 = .03$.

Figure S12

Descriptive statistics for the movement time in Experiment 2

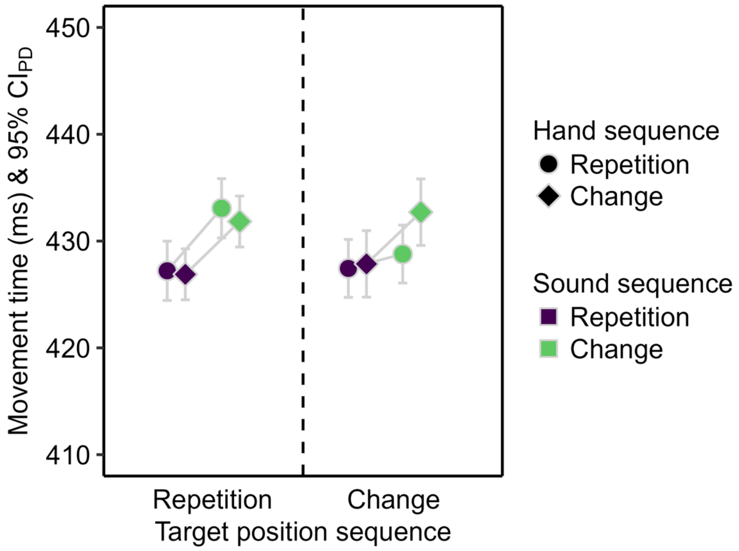

Note. Mean movement time in milliseconds (ms). Means are depicted as a function of sound repetition (purple) and change (green), hand repetition (circle) and change (diamond), and target position repetition (left) and change (right). The error bars are 95% confidence intervals of paired differences (CI_{PD}), visualizing two-tailed *t*-tests between sound sequences.

Exploratory re-analyses

We repeated the analyses described above for each dependent variable, this time, for a selected dataset where we controlled for the laterality of response sides. As described above for Experiment 1, we selected target position changes where the two targets appeared on the same side of the screen but at different positions for hand repetitions (48.5% trials excluded) and hand changes (50.9% trials excluded). All participants still contributed at least 10 observations in each design cell.

The response error was smaller for target position repetitions than changes (see Fig. S13), $F(1, 95) = 48.87$, $p < .001$, $\eta_p^2 = .34$, for hand repetitions and changes, $F(1, 95) = 7.75$, $p = .006$, $\eta_p^2 = .08$, and for sound changes than repetitions, $F(1, 95) = 4.53$, $p = .036$, $\eta_p^2 = .05$. The two-way interaction between target position and hand sequence was significant, $F(1, 95) = 4.16$, $p = .044$, $\eta_p^2 = .04$, because hand repetitions reduced the response error relative to hand changes only for target position repetitions,

$t(95) = 3.18$, $p = .002$, $d_z = 0.32$, not for target position changes, $t(95) = 1.31$, $p = .192$, $d_z = 0.13$. The remaining two-way interactions were not significant, $F < 1$. The three-way interaction was also not significant, $F(1, 95) = 1.85$, $p = .177$, $\eta_p^2 = .02$.

Figure S13

Descriptive statistics for the response error in Experiment 2

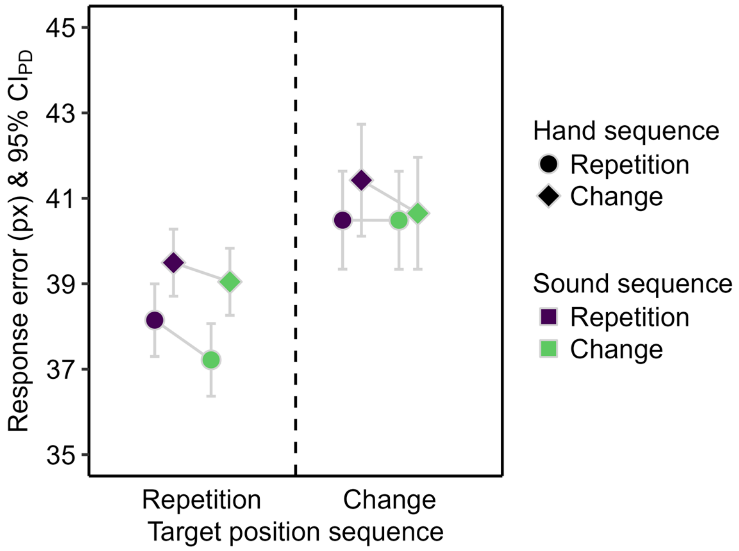

Note. Mean response error in pixels (px). Means are depicted as a function of sound repetition (purple) and change (green), hand repetition (circle) and change (diamond), and target position repetition (left) and change (right). The error bars are 95% confidence intervals of paired differences (CI_{PD}), visualizing two-tailed *t*-tests between sound sequences.

The response distance was shorter for target position repetitions than changes (see Fig. S14), $F(1, 95) = 14538.54$, $p < .001$, $\eta_p^2 > .99$, and for hand repetitions than changes, $F(1, 95) = 33.46$, $p < .001$, $\eta_p^2 = .26$. The main effect of sound sequence was not significant, $F < 1$. The two-way interaction between target position and hand sequence was significant, $F(1, 95) = 17.66$, $p < .001$, $\eta_p^2 = .16$, because the reduction of the response distance for hand repetitions compared to changes was significant for target position repetitions, $t(95) = 12.67$, $p < .001$, $d_z = 1.29$, but not for changes, $t(95) = 1.02$, p = .309, d = 0.10. The remaining two-way interactions and the three-way interaction were not significant, $F < 1$.

Figure S14

Descriptive statistics for the response distance in Experiment 2

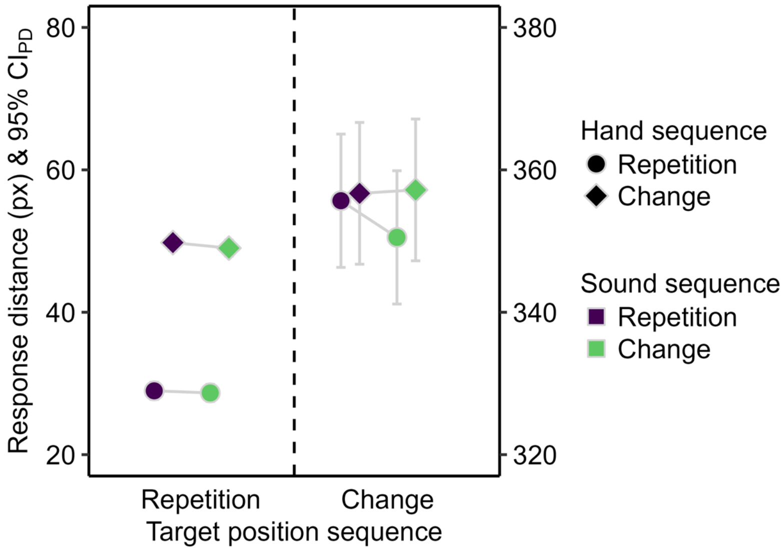

Note. Mean response distance in pixels (px). Means are depicted as a function of sound repetition (purple) and change (green), hand repetition (circle) and change (diamond), and target position repetition (left) and change (right). The error bars are 95% confidence intervals of paired differences (CI_{PD}), visualizing two-tailed *t*-tests between sound sequences. Changing the target position inevitably resulted in longer response distances than repeating the target position. Therefore, target position sequences are mapped to separate y-axes to facilitate comparison of sound and hand sequence effects.

The response direction change was smaller for target position repetitions than changes (see Fig. S15), $F(1, 95) = 75.43$, $p < .001$, $\eta_p^2 = .44$, and for hand repetitions than changes, $F(1, 95) = 309.54$, $p < .001$, $\eta_p^2 = .77$. The main effect of sound sequence was not significant, $F(1, 95) = 1.49$, $p = .225$, $\eta_p^2 = .02$. The interaction of target position and hand sequence was significant, $F(1, 95) = 52.44$, $p < .001$, $\eta_p^2 = .36$, because the reduction of the response direction change for hand repetitions compared to changes was larger for target position repetitions, $t(95) = 20.92$, $p < .001$, $d_z = 2.14$, than changes, $t(95) = 13.48$, $p < .001$, $d_z = 1.38$. The remaining two-way interactions and the three-way interaction were not significant, $F < 1$.

Figure S15

Descriptive statistics for the response direction change in Experiment 2

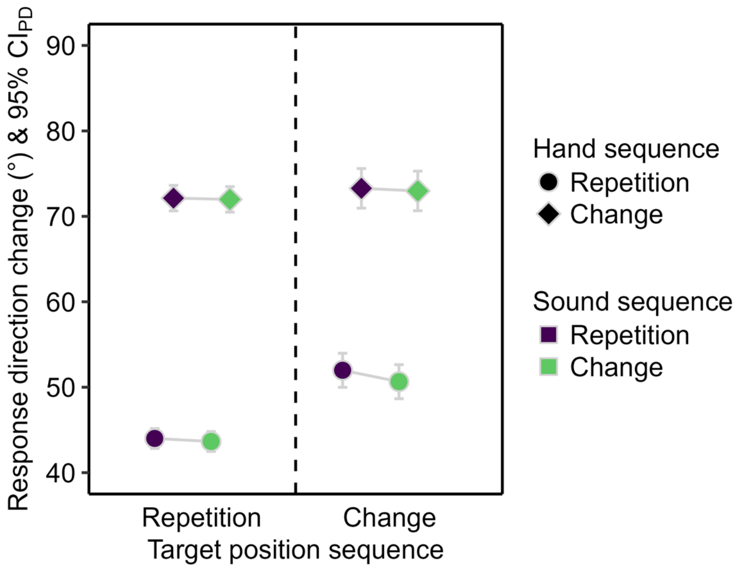

Note. Mean (absolute) response direction change in degrees (°). Means are depicted as a function of sound repetition (purple) and change (green), hand repetition (circle), and change (diamond), and target position repetition (left) and change (right). The error bars are 95% confidence intervals of paired differences (CI_{PD}), visualizing two-tailed *t*-tests between sound sequences.

The initiation time was shorter for target position repetitions than changes (see Fig. S16), $F(1, 95) = 163.61$, $p < .001$, $\eta_p^2 = .63$, for hand repetitions than changes, $F(1, 95) = 84.30$, $p < .001$, $\eta_p^2 = .47$, and for sound repetitions than changes, $F(1, 95) = 18.07$, $p < .001$, $\eta_p^2 = .16$. The interaction of target position and hand sequence was significant, $F(1, 95) = 66.78$, $p < .001$, $\eta_p^2 = .41$, indicating a larger benefit of hand repetitions relative to changes for target position repetitions, $t(95) = 10.76$, $p < .001$, $d_z = 1.10$, than changes, $t(95) = 6.68$, $p < .001$, $d_z = 0.68$. The two-way interaction of hand and sound sequence was also significant, $F(1, 95) = 63.68$, $p < .001$, $\eta_p^2 = .40$, because there was a significant benefit for sound repetitions relative to changes for hand repetitions, $t(95) = 10.60$, $p < .001$, $d_z = 1.08$, but a descriptive cost for changes, $t(95) = -1.93$, $p = .056$, $d_z = -0.20$. The remaining two-way and three-way interactions were not significant, $F < 1$.

Figure S16

Descriptive statistics for the initiation time in Experiment 2

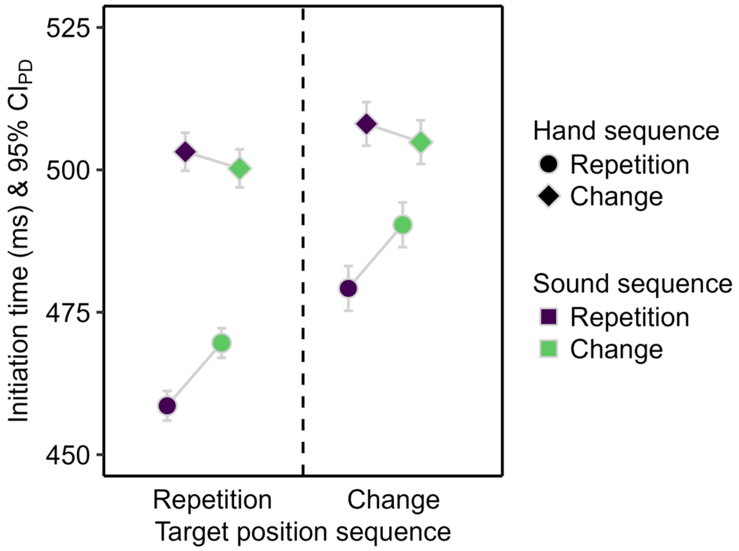

Note. Mean initiation time in milliseconds (ms). Means are depicted as a function of sound repetition (purple) and change (green), hand repetition (circle) and change (diamond), and target position repetition (left) and change (right). The error bars are 95% confidence intervals of paired differences (CI_{PD}), visualizing two-tailed *t*-tests between sound sequences.

In the movement time, the main effects of target position sequence and hand sequence were not significant (see Fig. S17), $F < 1$. The movement time was shorter for sound repetitions than changes, $F(1, 95) = 26.82$, $p < .001$, $\eta_p^2 = .22$. The two-way interactions were not significant, $F \leq 3.36$, $p \geq .3.36$, $\eta_p^2 \leq .03$. The three-way interaction of all factors was significant, $F(1, 95) = 4.45$, $p = .037$, $\eta_p^2 = .04$. The results for the same selection of target position repetitions are reported above (see *Secondary analyses*). For target position changes, the main effect of hand sequence was not significant, $F(1, 95) = 1.63$, $p = .205$, $\eta_p^2 = .02$. The main effect of sound sequence, $F(1, 95) = 6.85$, $p = .010$, $\eta_p^2 = .07$, and the interaction, $F(1, 95) = 4.06$, $p = .047$, $\eta_p^2 = .04$, were significant, indicating a benefit of sound repetitions over changes not for hand repetitions, $|t| < 1$, but for hand changes, $t(95) = 3.23$, $p = .002$, $d_z = 0.33$.

Figure S17

Descriptive statistics for the movement time in Experiment 2

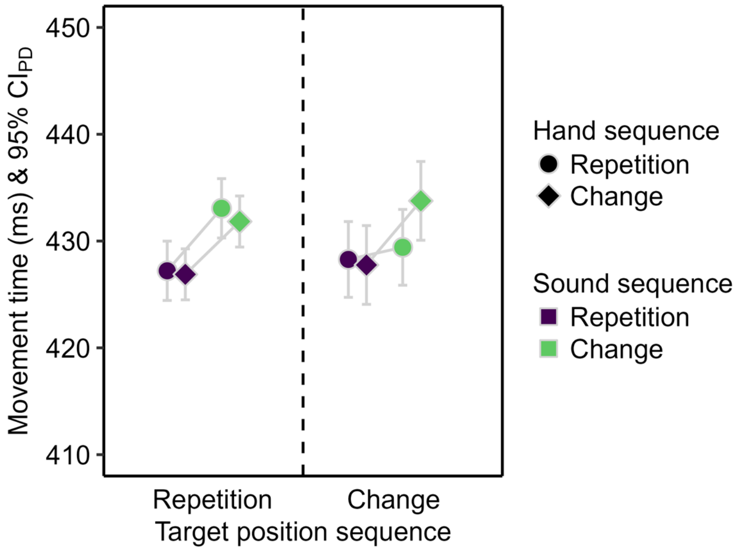

Note. Mean movement time in milliseconds (ms). Means are depicted as a function of sound repetition (purple) and change (green), hand repetition (circle) and change (diamond), and target position repetition (left) and change (right). The error bars are 95% confidence intervals of paired differences (CI_{PD}), visualizing two-tailed *t*-tests between sound sequences.

References

Foerster, A., Rothermund, K., Parmar, J. J., Moeller, B., Frings, C., & Pfister, R. (2021). Goal-based binding of irrelevant stimulus features for action slips. *Experimental Psychology, 68*(4), 206–213. https://doi.org/10.1027/1618-3169/a000525

R Core Team. (2023). *R: A Language and Environment for Statistical Computing* [Computer software]. https://www.R-project.org/

Schöpper, L.-M., Lappe, M., & Frings, C. (2022). Found in translation: The role of response mappings for observing binding effects in localization tasks. *Visual Cognition, 30*(8), 527–545. https://doi.org/10.1080/13506285.2022.2139033

Wiediger, M. D., & Fournier, L. R. (2008). An action sequence withheld in memory can delay execution of visually guided actions: The generalization of response compatibility interference. *Journal of Experimental Psychology: Human Perception and Performance, 34*(5), 1136–1149. https://doi.org/10.1037/0096-1523.34.5.1136
